# Supplementary material for: Efficient selection of knocked-in pluripotent stem cells using a dual cassette cellular elimination system
Source: Cell Rep Methods. 2023 Dec 11;3(12):100662. doi: 10.1016/j.crmeth.2023.100662 (PMC10753384; doi:10.1016/j.crmeth.2023.100662)
Supplement: Document S1. Figures S1–S4 and Tables S1 and S2 [file mmc1.pdf]

Cell Reports Methods, Volume *3*

Supplemental information

Efficient selection of knocked-in pluripotent stem cells using a dual cassette cellular elimination system

Koji Nakade, Satomi Tsukamoto, Kenichi Nakashima, Yuri An, Iori Sato, Jingyue Li, Yuzuno Shimoda, Yasuko Hemmi, Yoshihiro Miwa, and Yohei Hayashi

Figure S1

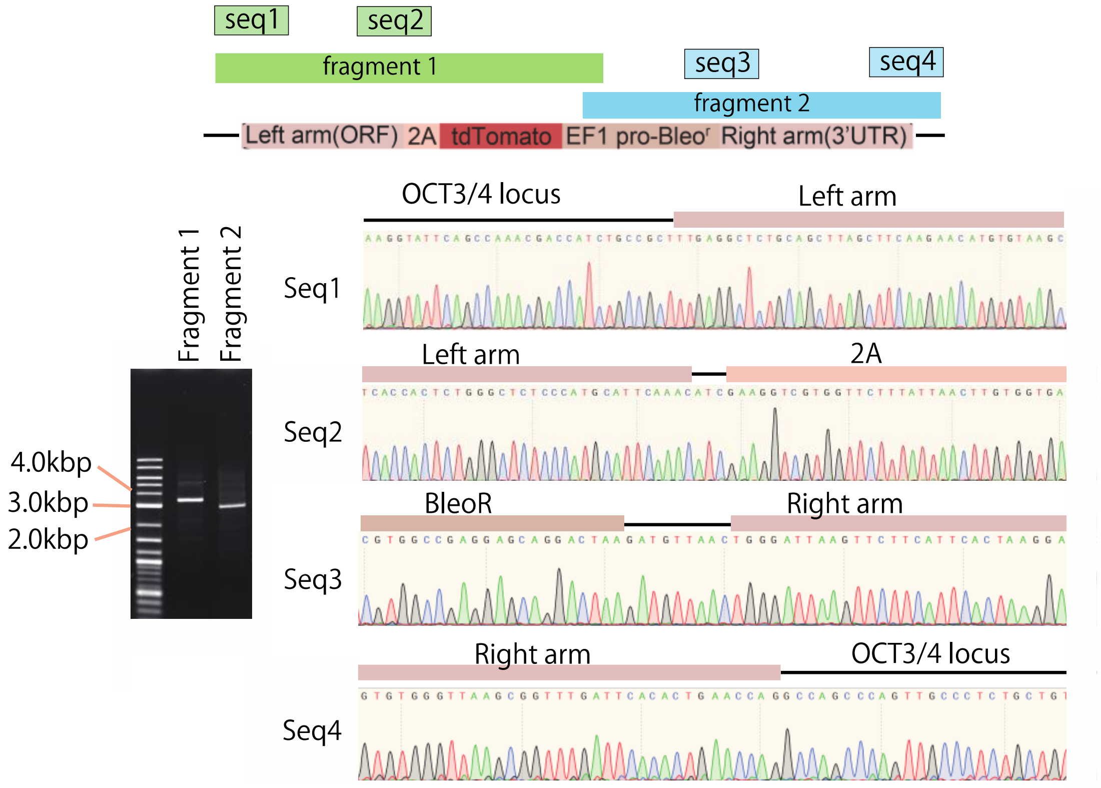

Figure S1. The representative sequencing schemes and results of *OCT4* genomic and knock-in alleles in a OCT3/4-TEZ(tk) clone, related to Figure 1.

Figure S2

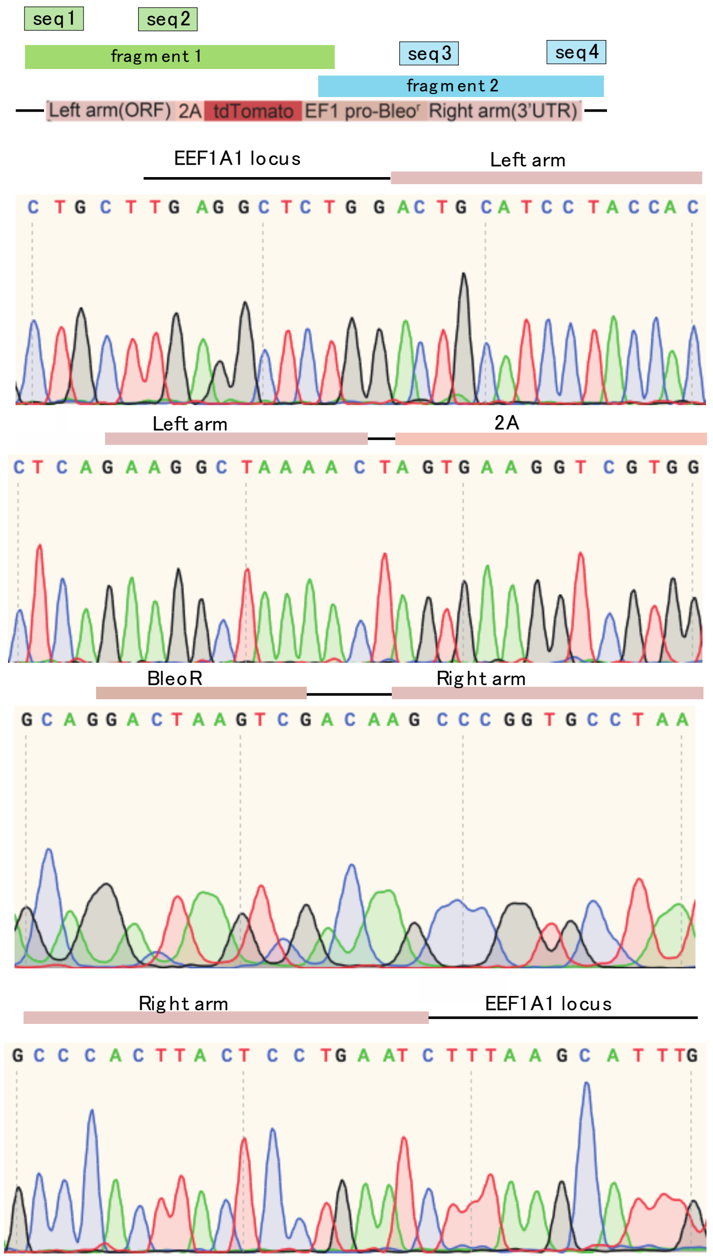

Figure S2. The representative sequencing schemes and results of *EEF1A1* genomic and knock-in alleles in a EEF1A1-TEZ knock-in clone, related to Figure 3.

Figure S3

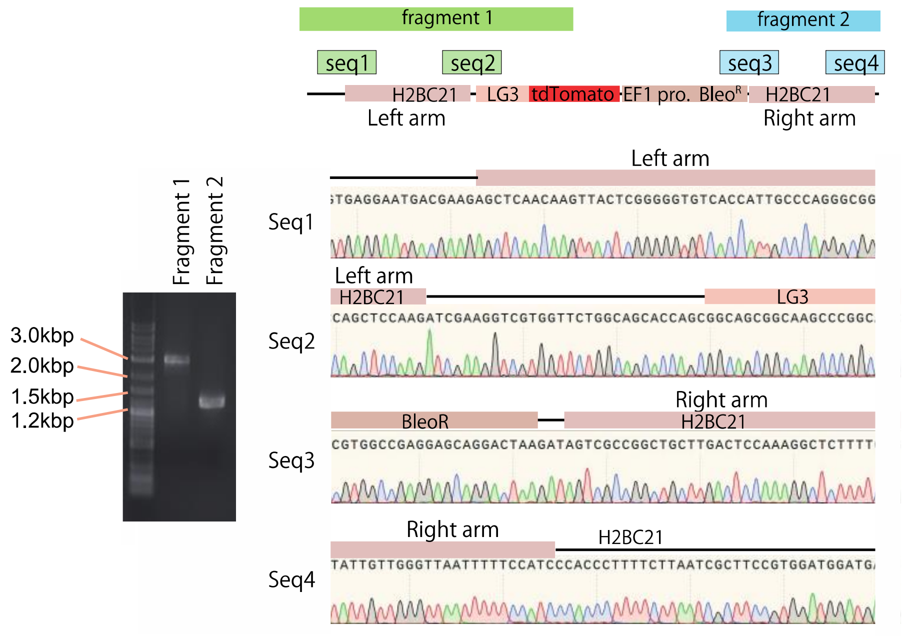

Figure S3. The representative sequencing schemes and results of *H2B* genomic and knock-in alleles in a H2B-TEZ knock-in clone, related to Figure 4.

Figure S4

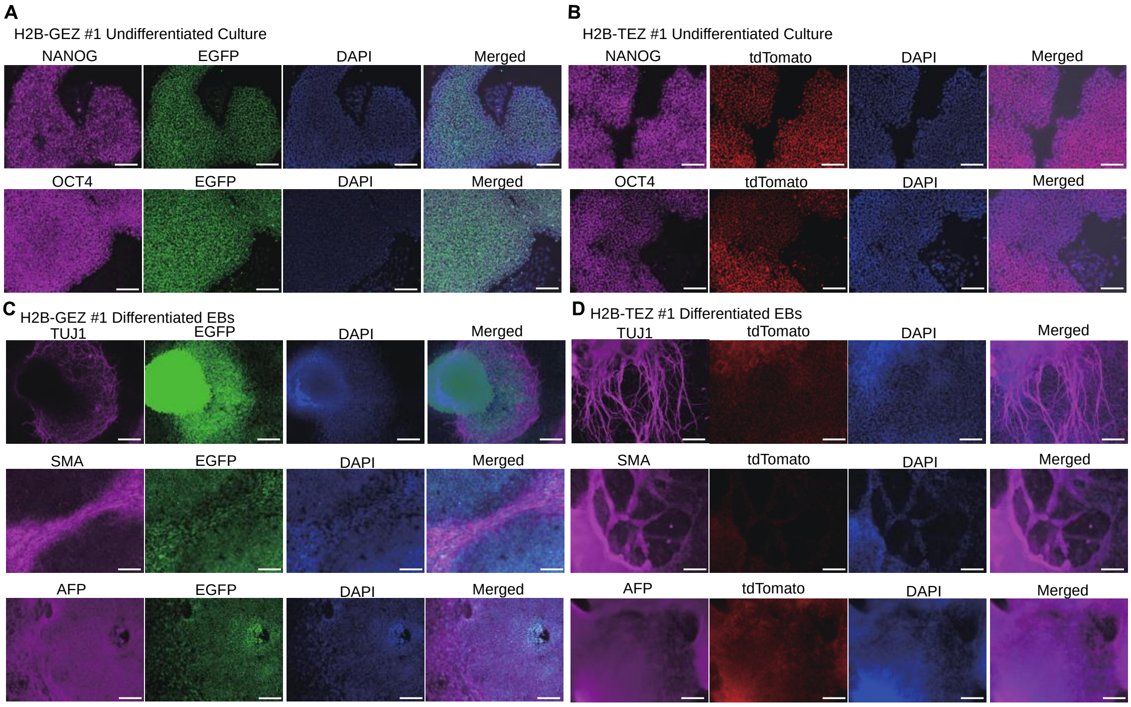

Figure S4. Self-renewal and pluripotency of H2B-GEZ #1 and H2B-TEZ #1, related to Figure 4. (A, B) Expression of self-renewal markers of hiPSCs, OCT3/4 and NANOG shown in purple. H2B-GEZ #1 and H2B-TEZ #1 were used. DAPI was used to stain nuclei (blue). EGFP or tdTomato were co-stained in green or red, respectively. Scale bars 100 μm. (C, D) Pluripotency in H2B-GEZ #1 and H2B-TEZ #1 was evaluated with EB formation assay. Immunocytochemistry of TUJ1 (ectoderm marker), alpha-SMA (mesoderm marker), and AFP (endoderm marker) in EB samples were shown in purple. DAPI was used to stain nuclei (blue). EGFP or tdTomato were co-stained in green or red, respectively. Scale bars, 100 μm.

Supplemental Table S1. The list of sequences of DNA primers or synthetic oligomers used in this study, related to STAR Methods.

Name	Sequence (5' -> 3')	Name	Sequence (5' -> 3')
gOCT3/4_F	CACCGAACTTAATCCCAAAAACCC	gOCT3/4_R	AAACGGGTTTTTGGGATTAAGTTC
gEEF1A1_F	CACCGAGAAACCTGSTGTGTTCCTT	gEEA1A1_R	AAACAAGGAACACACAGGTTTCTC
gISL1_F	CACCGATCATTGAATAGCATATAC	gISL1_R	AAACGTATATGCTATTCAATGATC
gH2B_F	CACCGACTCGAGCGAGCGAGCGCC	gH2B_R	AAACGGCGCTCGCTCGCTCGAGTC
gMYH7_F	caccTGTTACACAGGCTCCAGCAT	gMYH7_R	aaacATGCTGGAGCCTGTGTAACA
OCT3/4 LA-f	ATGGAAAAACGCCAGTTGAGGCTCTGCAGCTTA	OCT3/4 RA-r	AAAACGACGGCCAGTGCTGGTTCAGTGTGAATC
p15AoriF	TATAGTCCTGTCGGGTTTCG	OCT3/4 LA-r	AGAACCACGACCTTCGATGTTTGAATGCATGGGAGAG
OCT3/4 RAf	AAGCCCGGTGCCTAATCTAGATTCTAGGAATGGGGGACAGGG	p15AoriR	AACCCGACAGGACTATA
OCT3/4KI_Pr399	CAACTGCGTGCACTTCGTGG	OCT3/4KI_chkRA-r2	AGCAGAGGGCAACTGGGCTG
OCT3/4WT+KI_RAf-HpaI	GTTAACTGGGATTAAGTTCTTCATTCACTA		
EEF1A1 LA-f	ATGGAAAAACGCCAGCCGTAAGGATGGCAATGCCAGT	EEF1A1 RA-r	GACGGCCAGTGAATTGGAGTAAGTGGGCTATTACACCTG
EEF1A1 RA-f	AAGCCCGGTGCCTAATCTAGATGTGTTCCTTTGGTCAACA	EEF1A1 LA-r	AGAACCACGACCTTCACTAGTTTTAGCCTTCTGAGCTTTC
2Af-1-16	GAAGGTCGTGGTTCT	Bleo-R inf-puro	TTAGGCACCGGGCTTGTCGACTTAGTCCTGCTCCTCG
H2B LA-f	ATGGAAAAACGCCAGCTCAACAAGTTACTCGGGGGTGTCA	H2B LA-r	TCCTCGGTACCAGAACCACGACCTTCGATCTTGGAGCTGGTGTACTTGGT
H2B RA-f	GTTCTGGTACCGAGGAGCAGGACTAAGATAGTCGCCGGCTGCTTGACTCCA	H2B RA-r	AAAACGACGGCCAGTGGATGGAAAAATTAACCCAACAATAT
M13RV	CAGGAAACAGCTATGAC	tdTom-r	CTCCTCGCCCTTGCTCACCAT
chk*ISL1* LA-f	TACCTGGAGGGCATTTGCTTT	*ISL1* LA-r	AGAACCACGACCTTCTGCCTCAATAGGACTGGCTAC
chk*ISL1* RA-r	GGCGCTTTGATTTCACATA	BleoR-f	GCACTTCGTGGCCGAGGAGCAGGAC
GFP-f	ATGGTGAGCAAGGGCGA	GFP-r	GGGTCGACCTTGTACAGCTCGTCCA
*H2BC21*_genotype_F	AGGGCGGGACTTGGCAGTTTAGAG	*H2BC21*_genotype_R	GTAAGTGCCTCGCAGTGACCTGGG
hsv-tk_genotype_F	TGGCTTCGTACCCCTGCCATCAAC	hsv-tk_genotype_R	AGCACCTGCCAGTAAGTCATCGGC
hsv-tk_F3	GCTTGCCAATACGGTGCGGT	hsv-tk_Pr949	CTTTTTATTGCCGTCATAGCG
ISL1 RA-f3	CCCTGTTGGAGAAAGTGGGA	*ISL1* RA-r3	CCACCTTCCTGAAAGGACTCT
MYH7 LA-f	tatagcatgaacagtGTCCAAGTTCCGCAAGGTGCAG	*MYH7 LA-r*	agaaccacgaccttcCTCCTCATTCAAGCCCTTTTGA
MYH7 RA-f	aagcccggtgcctaaCTGGAGCCTGTGTAACAGCT	*MYH7 RA-r*	aaaacgacggccagtACTCTTTCCTCCACCTCCTGGCC
chkMYH7 LA-r	CAGATCAAGATGTGGCAAAGCT	*chkMYH7 RA-r*	ACTCCAAAGGGGCTGAACTTG
ckOct3KI-LarmoutF	ctcaccctgggggttctatttggtg	*ckOct3KI-RarmoutR*	TAAGGGAAAACAGATCTATTGCCAT
EF1a-M_R	ACTTCCAACCCGAAGCTCG	*BGH_F2*	CTGTGCCTTCTAGTTGCCAG
tdTomato hindge-r	gttgttgtcctcggaggaggc	*ZeoR_F1*	ACGAGCTGTACGCCGAGTGG

Supplemental Table S2. The list of DNA plasmids used in this study, related to STAR Methods.

Plasmid Name	Reference number
pCRtk2x2-ISL1-TEZ	RDB18669, RIKEN BRC
pCRtk2x2NN	RDB18670, RIKEN BRC
pX330-gIsl1	RDB18671, RIKEN BRC
pUC-TEZ	RDB18672, RIKEN BRC
pUCtk2x2	RDB19569, RIKEN BRC
pX459-gEEF1A1	RDB19570, RIKEN BRC
pUCtk2x2-EEF1-TEZ	RDB19571, RIKEN BRC
pUCtk2x2-OCT3/4-TEZ	RDB19572, RIKEN BRC
pUC-2xLG3-GEZ	RDB19573, RIKEN BRC
pUC-2xLG3-TEZ	RDB19574, RIKEN BRC
pUCtk2x2-UAS	RDB19575, RIKEN BRC
pUC2x2Tk-H2B	RDB19576, RIKEN BRC
pUC2x2Tk-H2B-GEZ	RDB19577, RIKEN BRC
pUC2x2Tk-H2B-TEZ	RDB19578, RIKEN BRC
pUCtk2x2-MYH7-TEZ	RDB20120, RIKEN BRC
pX330-gH2B	RDB19579, RIKEN BRC
pX330-gOCT3/4	RDB19580, RIKEN BRC
pX330-gMYH7	RDB20121, RIKEN BRC
pUC-OCT3/4-TEZ	RDB19586, RIKEN BRC
pX330	#42230, Addgene
pX459	#62988, Addgene
pEGFP-N1	#6085-1, Clontech
