# Supplementary material for: Efficient selection of knocked-in pluripotent stem cells using a dual cassette cellular elimination system
Source: Cell Rep Methods. 2023 Dec 11;3(12):100662. doi: 10.1016/j.crmeth.2023.100662 (PMC10753384; doi:10.1016/j.crmeth.2023.100662)
Supplement: Document S2. Article plus supplemental information [file mmc2.pdf]

Cell Reports Methods

Article

Efficient selection of knocked-in pluripotent stem cells using a dual cassette cellular elimination system

Graphical abstract

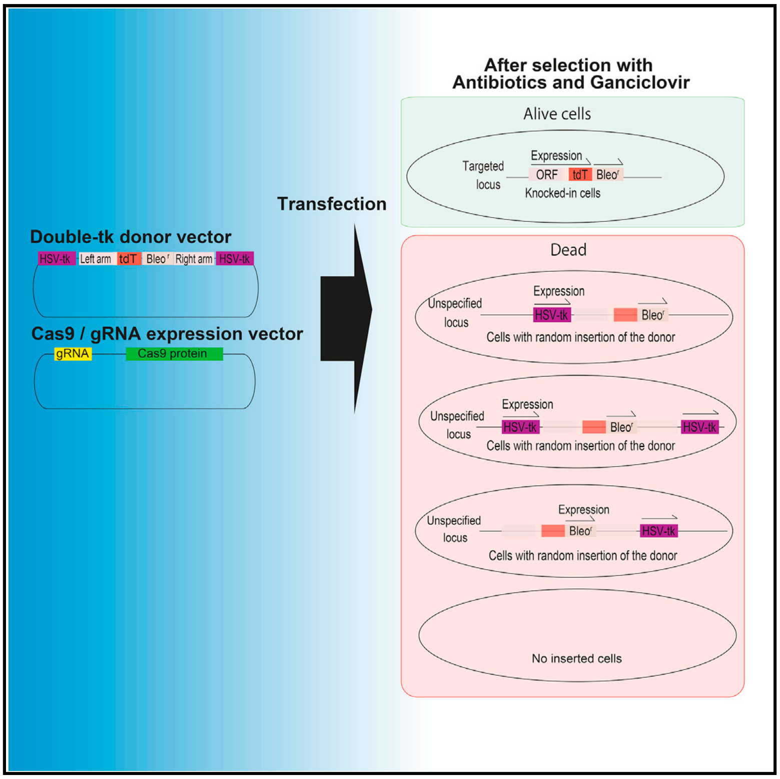

Highlights

- We develop a "double-tk donor vector system" for selection of HDR-mediated knockins
- This system improves enrichment of knocked-in cells after transduction
- Using this system, we efficiently generate fluorescent reporter knockin hiPSCs

Authors

Koji Nakade, Satomi Tsukamoto, Kenichi Nakashima, ..., Yasuko Hemmi, Yoshihiro Miwa, Yohei Hayashi

Correspondence

koji.nakade@riken.jp (K.N.),
yohei.hayashi@riken.jp (Y.H.)

In brief

To improve the efficiency of gene knockin based on homology-directed repair, Nakade et al. develop a cell selection method based on donor vectors carrying double HSV-tk cassettes. Using the method, they demonstrate efficient generation of a variety of fluorescent reporter knockin hiPSCs.

Nakade et al., 2023, Cell Reports Methods 3, 100662
December 18, 2023 © 2023 The Authors.
https://doi.org/10.1016/j.crmeth.2023.100662

CellPress

Cell Reports Methods

CellPress
OPEN ACCESS

Article

Efficient selection of knocked-in pluripotent stem cells using a dual cassette cellular elimination system

Koji Nakade,[1,*] Satomi Tsukamoto,[2] Kenichi Nakashima,[1] Yuri An,[2] Iori Sato,[2] Jingyue Li,[2] Yuzuno Shimoda,[2,3] Yasuko Hemmi,[2] Yoshihiro Miwa,[1] and Yohei Hayashi[2,3,4,*]

[1]Gene Engineering Division, BioResource Research Center, RIKEN, 3-1-1 Koyadai, Tsukuba, Ibaraki 305-0074, Japan

[2]iPS Cell Advanced Characterization and Development Team, BioResource Research Center, RIKEN, 3-1-1 Koyadai, Tsukuba, Ibaraki 305-0074, Japan

[3]School of Integrative and Global Majors, University of Tsukuba, 1-1-1 Tennodai, Tsukuba, Ibaraki 305-8577, Japan

[4]Lead contact

*Correspondence: koji.nakade@riken.jp (K.N.), yohei.hayashi@riken.jp (Y.H.)

https://doi.org/10.1016/j.crmeth.2023.100662

MOTIVATION The efficiency of gene knockin based on homology-directed repair (HDR) can be low, particularly in stem and primary cells. In addition, targeting a specific position in the genome can generally result in cells with untargeted insertion of the donor vector at non-specific locations or in carrying a cytoplasmic residue of the donor vector. Thus, we developed an improved method to select knocked-in cells efficiently based on donor vectors carrying double thymidine kinase of herpes simplex virus (HSV-tk) cassettes.

SUMMARY

Although recent advances in genome editing technology with homology-directed repair have enabled the insertion of various reporter genes into the genome of mammalian cells, the efficiency is still low due to the random insertion of donor vectors into the host genome. To efficiently select knocked-in cells without random insertion, we developed the "double-tk donor vector system," in which the expression units of the thymidine kinase of herpes simplex virus (HSV-tk) are placed on both outer sides of homology arms. This system is superior in enriching knocked-in human induced pluripotent stem cells (hiPSCs) than conventional donor vector systems with a single or no HSV-tk cassette. Using this system, we efficiently generated fluorescent reporter knockin hiPSCs targeting *POU5F1* (*OCT3/4*), *EEF1A1*, *H2BC21* (*H2B clustered histone 21*), *ISL1*, and *MYH7* genes. These results indicate that the double-tk donor vector system enables efficient selection of knocked-in hiPSCs carrying reporter proteins.

INTRODUCTION

Various methods for differentiating pluripotent stem cells (PSCs) into specific cell types have been developed, and differentiated cells derived from PSCs have been widely used for modeling cellular transplantation therapies and evaluating drug candidates, as well as for basic biology.[1–3] Methods involving cell destruction, such as immunocytochemistry and flow cytometry for detecting marker protein expression, quantitative RT-PCR, or RNA sequencing (RNA-seq) for detecting gene expression patterns, are usually required to confirm that PSCs have differentiated into the anticipated cell type. To avoid such cell destruction and monitor cellular status in living cells, integrating fluorescent or luminescent reporter genes with genes of interest endogenously or exogenously is required for cellular visualization. In particular, knocking in a gene encoding a fluorescent protein to the end of various differentiation marker genes of PSCs using genome editing techniques enables precise monitoring of gene expression and selection of specific cell types. Knocking in exogenous DNA into the host genome can be achieved via homology-directed repair (HDR) induced by zinc-finger nucleases (ZFNs), TAL effector nucleases (TALENs), and clustered regularly interspaced short palindromic repeats (CRISPR)-CRISPR-associated protein 9 (Cas9) nucleases. The HDR knockin method using CRISPR-Cas9 is generally performed by co-transfecting the Cas9 nuclease/guide RNA (gRNA) expression vector and a donor vector.[4–7]

The efficiency of knocking in based on HDR is generally low, particularly in stem and primary cells. Targeting a specific position in the genome by co-transfecting the Cas9 nuclease/gRNA expression vector and a donor vector primarily results

This is an open access article under the CC BY license (http://creativecommons.org/licenses/by/4.0/).

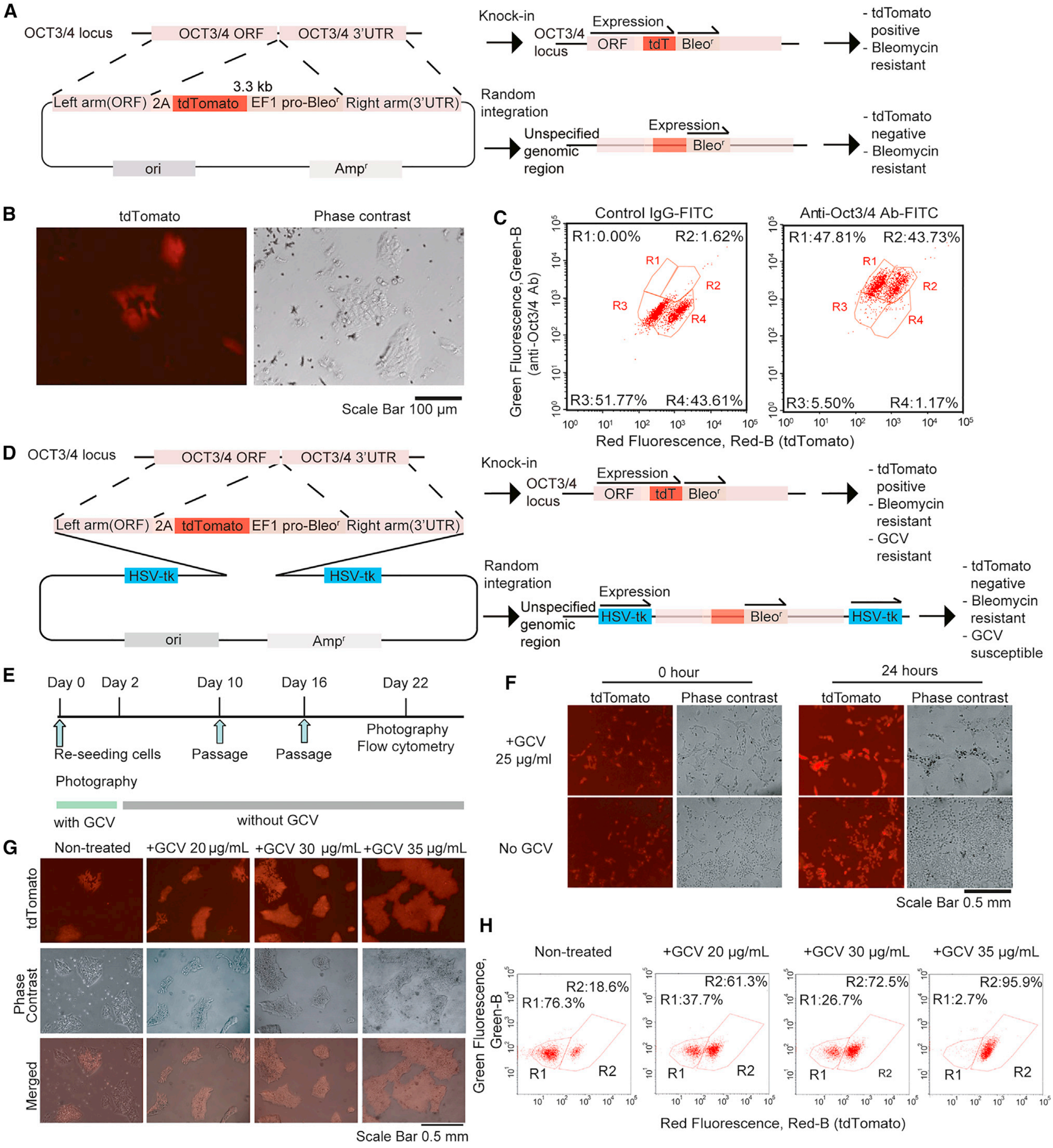

Figure 1. Knocking in to *OCT3/4* locus with double-tk donor vector

(A) Structure of the donor vector pUC OCT3/4-TEZ.

(B) Fluorescent and phase-contrast images of OCT3/4-TEZ bulk culture. After transfection of hiPSCs with pUC OCT3/4-TEZ and pX330-gOCT3/4, knocked-in hiPSCs were selected by zeocin.

(C) Flow cytometry analysis of the OCT3/4-TEZ bulk culture. The cells were fixed, permeabilized, and stained with rabbit anti-OCT3/4 antibody and donkey anti-rabbit immunoglobulin G-fluorescein isothiocyanate (IgG-FITC) as the primary and secondary antibodies, respectively.

(D) Schematics of knockin strategy using double-tk donor vectors. When the constructed vector is randomly inserted into the genome, the cells become sensitive to ganciclovir (GCV).

(legend continued on next page)

in cells carrying these vectors inserted in a non-specific location of genomic DNA or remaining in the cytoplasm transiently. For example, the trial for knockin of the *GFP* gene to β-actin and the *LMNB1* gene locus generated 8.5% and 11.5% of knocked-in human induced PSCs (hiPSCs), respectively, even after antibiotic selection.[8] Although some studies have developed techniques to boost HDR by modulating the cell cycle,[9,10] DNA repair pathways,[11,12] or p53-mediated DNA damage responses,[13,14] non-specific gene insertions are unavoidable because the methods of producing knocked-in cells are partly similar to the process of producing stable transformants. Cloning knocked-in cells is an effective way to selectively acquire targeted insertion of a donor vector; however, it is tedious and challenging to obtain knocked-in cells without random insertions when the knockin efficiency is low. Furthermore, it is difficult to determine and select the correct knocked-in cells when differentiation marker genes are targeted unless a genotyping analysis is performed. Indeed, the knock in of the *EGFP* gene to the *PITX3* gene, which is expressed in some differentiated cell types, generated only 8%–11% knocked-in clones of human embryonic stem cells (hESCs) and hiPSCs even after antibiotic selection.[15]

Negative selection based on the cellular suicide system has been employed to enrich knocked-in cells with homologous recombination. In particular, ganciclovir (GCV) treatment has been widely used to kill cells expressing the thymidine kinase of herpes simplex virus (HSV-tk) from the outer side of the homology arms in donor vectors.[16,17] Gene-edited cells with randomly incorporated donor vectors harbor HSV-tk and therefore can be eliminated by the addition of GCV.[18,19] Although this method is widely used, the efficiency of negative selection in mammalian PSCs with a single HSV-tk expression cassette still poses challenges.[20,21]

In this study, we developed a method to selectively grow knockin cells using a cellular suicide system based on a double-tk donor vector. This donor vector has the expression units of the HSV-tk placed on both outer sides of homology arms, which are approximately 1 kb upstream and downstream of the end of the open reading frame (ORF) of the gene of interest. And a DNA fragment encoding fluorescent protein was inserted via linker peptides instead of the stop codon, followed by an antibiotic-resistance gene driven by the EF1 promoter inserted downstream of the fluorescent protein-encoding gene to select gene-transfected cells. During cell division, homologous recombination of the two homology arms occurs at the site of genomic DNA cleavage by Cas9 nuclease and gRNA, and the fluorescent gene linked to the arms is incorporated. While cells with donor vectors carrying HSV-tk at non-target loci were eliminated in the presence of GCV, the knocked-in cells grew selectively.

RESULTS

Inefficient knocking in to *OCT3/4* locus with conventional donor vector system

We first selected active genes for knockin targets, including the undifferentiated marker gene *OCT3/4* (*POU5F1*),[22] as models to facilitate the evaluation of knockin efficiency via fluorescence in hiPSCs. To test the efficacy of knockin with a conventional donor vector system, we transfected hiPSCs with the donor vector pUC-OCT3/4-TEZ, which contained 2A self-cleaving peptide and tdTomato sequences followed by the EF1 alpha promoter-driven bleomycin-resistance gene surrounded by the homology arms of the *OCT3/4* sequences, and the expression vector for Cas9 nuclease and gRNA to cleave the 3′ end of the *OCT3/4* ORF (Figure 1A). After selecting transduced cells with zeocin, a bleomycin derivative, some hiPSCs expressed tdTomato fluorescence; however, cells that did not express tdTomato but had the characteristic shape of hiPSCs were also observed (Figure 1B). The resulting hiPSCs were examined for fluorescence and OCT3/4 protein expression with flow cytometry. We found that only 44% of hiPSCs were tdTomato positive, while approximately 92% of the whole population of hiPSCs were positive for the OCT3/4 protein stained with anti-OCT4 and fluorescence-labeled secondary antibodies (Figure 1C). These results raised the possibility that the positive selection of knocked-in cells by bleomycin-resistant genes and antibiotics treatment was insufficient, implying that many of the resistant cells might carry the donor vector with the resistance gene sequence that was either randomly inserted into unexpected genomic loci or transiently expressed by the donor vector.

Efficient selection of OCT3/4-tdTomato knocked-in cells with double-tk donor vectors

In the case of knockin by HDR, the DNA sequence of the donor vector outside the homology arm was not incorporated into the genome. Alternatively, the sequences of the donor vector may be cut off at an arbitrary position and randomly inserted into a non-specific site in the genome. In addition, the expression of antibiotic-resistance genes from the residual vector in the cytoplasm may transiently produce antibiotic-resistant cells without knockin or random integration (Figure 1A). We hypothesized that inserting inducible suicide gene cassettes outside of the homology arm on the donor vector would lead to the efficient elimination of untargeted inserted cells and that the combination of antibiotic selection would enrich the knocked-in cells. In particular, having two copies of the suicide gene cassettes outside of the homology arm on the donor vector should enrich these knocked-in cells efficiently for negative selection, even through vector DNA cleavage accompanied by genome editing. The expression of a suicide gene, the HSV-tk, causes cellular suicide

(E) Selection of OCT3/4-tdTomato knocked-in cells by GCV/double-tk system. The experimental scheme for the GCV selection of OCT3/4-tdTomato knocked-in hiPSCs.

(F) Time course imaging of GCV treated or non-treated hiPSCs. The bulk culture was grown in the presence or absence of 25 μg/mL GCV, and their fluorescence and phase-contrast images were photographed after 24 h. Scale bar, 0.5 mm.

(G) Microscope images of OCT3/4-TEZ(tk) hiPSCs selected with GCV. Scale bar, 0.5 mm.

(H) Flow cytometric analysis of OCT3/4-TEZ(tk) hiPSCs selected with GCV. The tdTomato-negative and -positive cells were gated in R1 and R2, respectively. The percentages of the cells in the gates are shown.

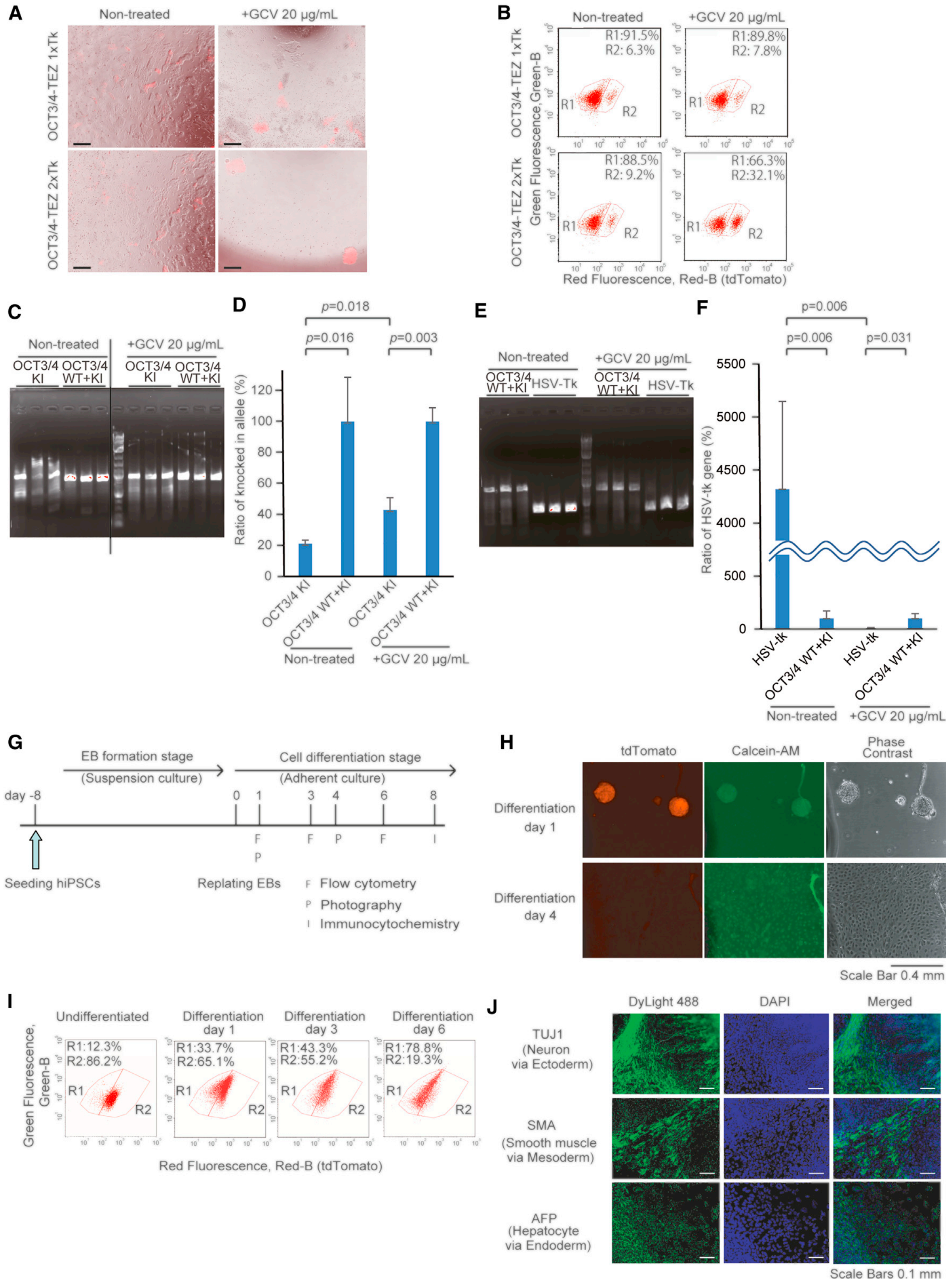

Figure 2. Characterization of hiPSCs generated with double-tk donor vectors carrying OCT4-TEZ

(A) Microscope images of hiPSCs transfected with single-tk or double-tk donor vectors and selected with or without GCV. Scale bar, 0.5 mm.

(B) Flow cytometric analysis of hiPSCs transfected with single-tk or double-tk donor vectors and selected with GCV. The tdTomato-negative and -positive cells were gated in R1 and R2, respectively. The percentages of the cells in the gates are shown.

(legend continued on next page)

upon GCV treatment.[16,17] Thus, we developed a backbone vector with two copies of HSV-tk expression units on both sides of the homology arm sequence (Figure 1D).

To test the efficacy of the double-tk donor vector, we transfected hiPSCs with the donor vector, which targets the C terminus of OCT3/4 gene, and the expression vector for Cas9 nuclease and gRNA to cleave the 3′ end of the *OCT3/4* ORF (Figure 1E). After selection with zeocin, the surviving cells were reseeded and incubated with GCV at 20, 30, and 35 μg/mL for 2 days. Non-fluorescent cells were largely dead after 1 day of treatment (Figure 1F). Higher concentrations of GCV enriched tdTomato-positive cells but also increased the number of dead cells, including tdTomato-positive cells. After GCV treatment, the medium was changed every 2 days, and the cells were passaged on days 10 and 16 of the culture. After 22 days of treatment with GCV, fluorescence imaging and fluorescence-activated cell sorting (FACS) analysis of the surviving cells were performed. The results showed that 61.3% and 72.5% of the cells were positive for tdTomato after treatment with 20 and 30 μg/mL GCV, respectively, whereas 18.6% of the untreated cells were positive for tdTomato (Figures 1G and 1H). With 35 μg/mL GCV, 96% of the cells were positive for tdTomato, and these cells were from a single surviving colony. These results indicate that knocked-in cells generated with the double-tk donor vector can be efficiently selected by exposing the cells to appropriate concentrations of GCV. In a different experiment, we compared this double-tk donor vector with a single-tk donor vector in terms of knockin efficiency. In hiPSCs electroporated with the double-tk donor vector, with and without GCV treatment, 9.2% and 32.1% of the cells were tdTomato positive, respectively (Figures 2A and 2B). In contrast, hiPSCs electroporated with the single-tk donor vector, with and without GCV treatment, resulted in 6.3% and 7.8% tdTomato-positive cells, respectively. These results indicate that double-tk donor vectors more efficiently enriched knocked-in hiPSCs than single-tk donor vectors.

Next, we isolated hiPSC clones from the tdTomato-positive cell population under GCV-treated or non-treated conditions. Genotyping PCR results showed that hiPSC clones from the GCV-treated population carried a significantly higher ratio of knockin alleles than those from the untreated population (Figures 2C and 2D). In addition, hiPSC clones from the GCV-treated population carried a significantly lower HSV-tk sequence ratio than those from the untreated population (Figures 2E and 2F). There were no detectable mutations around knockin sequences in these hiPSC clones (Figure S1). These results indicate that GCV treatment efficiently enriched knockin cells and eliminated cells randomly inserted with donor vectors after genome editing with double-tk donor vectors.

To examine whether GCV treatment maintained hiPSC pluripotency, we formed embryoid bodies (EBs) from OCT3/4-TEZ(tk) cells treated with 20 μg/mL GCV (OCT3/4-TEZ(tk) GCV20). OCT3/4-TEZ(tk) GCV20 hiPSCs were grown on an untreated dish for 7 days to form EBs, and then the EBs were transferred to a tissue culture dish for 4 days for fluorescence imaging (Figure 2G). Before imaging, calcein-AM was added to the medium to stain viable cells. After 7 days of suspension culture, OCT3/4-TEZ(tk) GCV20 hiPSCs formed EBs (Figure 2H, day 1). Under subsequent adherent culture conditions, the red fluorescence of the EB-derived cells decreased with cell differentiation and disappeared by day 4 (Figure 2H, day 4). The cells cultured under adherent conditions for 1, 2, or 6 days were harvested and analyzed with flow cytometry. The EB-derived cells began losing their red fluorescence due to cell differentiation on the first day after attachment to the dish surface, and the red fluorescence disappeared by day 6 (Figure 2I). After 8 days of differentiation, the EBs contained cells positive for TUJ1 (TUBB3 [tubulin, β 3 class III]), smooth muscle actin (SMA), and alpha-fetoprotein (AFP), which indicated that these cells differentiated into three germ layers (Figure 2J). These results indicated that OCT3/4-TEZ(tk) hiPSCs treated with GCV maintained pluripotency and that the expression of tdTomato knocked in at the *OCT3/4* locus acted as an indicator of the undifferentiated state.

Efficient selection of EEF1A1-tdTomato knocked-in cells

EEF1A1 encodes a subunit of the elongation factor 1 complex, which delivers aminoacylated tRNAs to the ribosome.[23] It is one of the housekeeping genes constitutively expressed in many types of cells, including hiPSCs. We used a knockin system for *EEF1A1* as a second gene to assess the efficiency of GCV selection. The hiPSC line 1383D6 was transfected with the double-tk donor vector targeting EEF1A1 and the Cas9 nuclease/gRNA expression vector and selected in the presence

(C) Representative data of genotyping PCR of OCT3/4 wild-type (WT) and WT plus knockin (WT + KI) alleles in OCT4-TEZ hiPSC clones obtained from GCV-treated or non-treated conditions.

(D) Quantification of allele frequency (%) from genotyping PCR of OCT3/4 WT and WT + KI alleles in OCT4-TEZ hiPSC clones obtained from GCV-treated or non-treated conditions. The results are shown as mean with SE (n = 3). p values were calculated from Student's t test.

(E) Representative data of genotyping PCR of OCT3/4 WT + KI allele and exogenous HSV-tk gene in OCT4-TEZ hiPSC clones obtained from GCV-treated or non-treated conditions.

(F) Quantification of allele frequency (%) from genotyping PCR of OCT3/4 WT + KI allele and exogenous HSV-tk gene in OCT4-TEZ hiPSC clones obtained from GCV-treated or non-treated conditions. The results are shown as mean with SE (n = 3).

(G) The experimental scheme for embryoid body (EB) formation and differentiation assay.

(H) Photograph of the cells differentiating from EBs in a cell culture dish. Red and green fluorescence are derived from tdTomato (undifferentiated cell indicator) and calcein-AM (living cell indicator), respectively. Scale bar, 0.4 mm.

(I) Flow cytometric analysis of the differentiating cells from EBs. The differentiating cells on the tissue culture dish were detached on the indicated day and analyzed by flow cytometry. The tdTomato-negative and -positive cells were gated in R1 and R2, respectively. The percentages of the cells in the gates are shown.

(J) Immunocytochemistry of an ectodermal marker, TUJ1 (TUBB3 [tubulin, β 3 class III]); a mesodermal marker, SMA (smooth muscle actin); and an endodermal marker, AFP (alpha-fetoprotein). The nuclei were stained with DAPI. Scale bar, 0.1 mm.

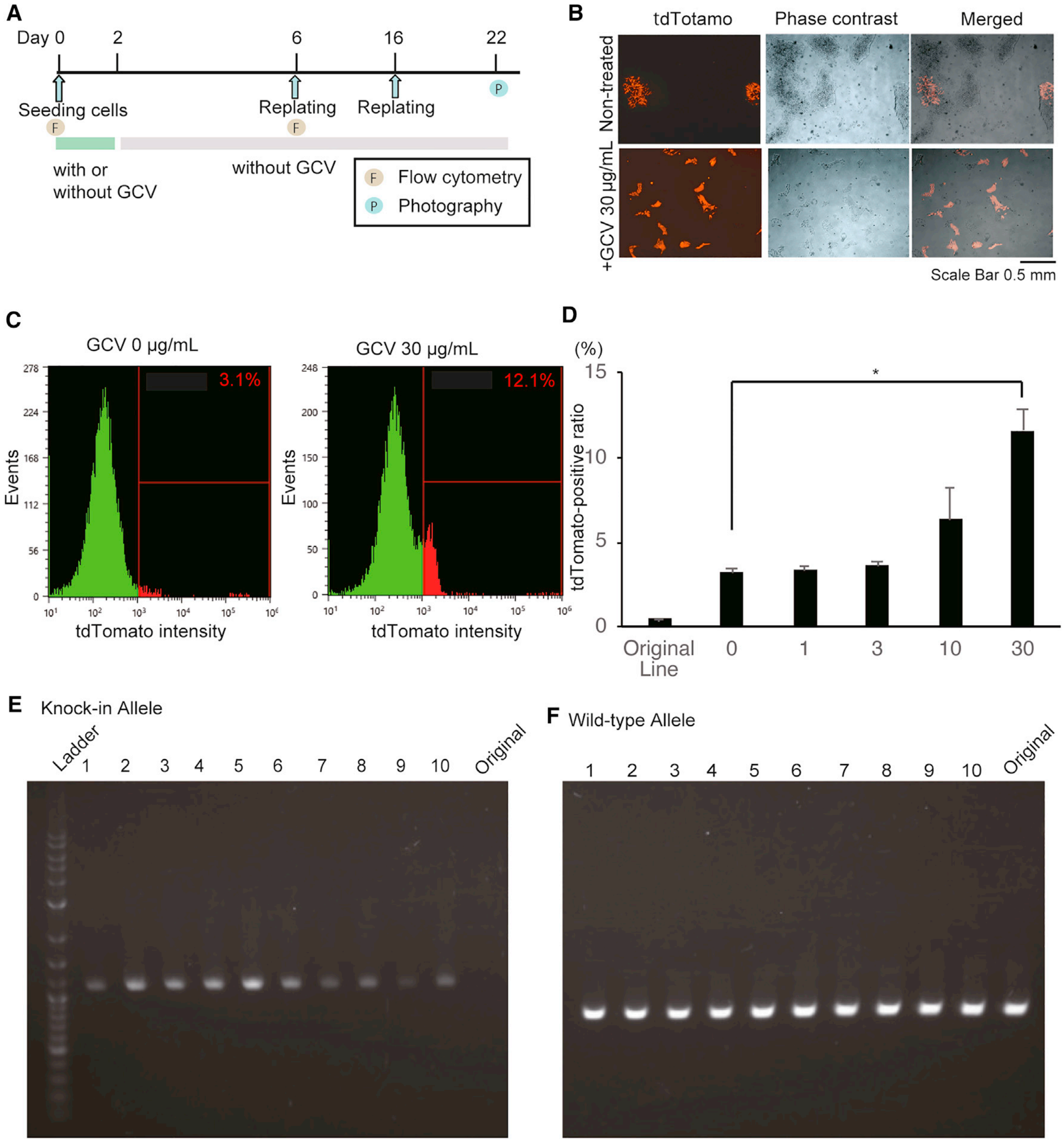

Figure 3. Selection of EEF1A1-tdTomato knocked-in cells by GCV/double-tk system

(A) The experimental scheme for the GCV selection of EEF1A1-tdTomato knocked-in hiPSCs (EEF1A1-TEZ iPSCs).

(B) Microscope images of EEF1A1-TEZ hiPSCs selected with 30 μg/mL GCV. Fluorescence and phase-contrast images of the cells after 20 days post-addition of GCV are shown. Successfully knocked-in cells are tdTomato positive upon EEF1A1 expression. Scale bar, 0.5 mm.

(C) Representative images of flow cytometric analysis on EEF1A1-TEZ iPSCs selected with GCV at concentrations of 0 or 30 μg/mL. The percentages of the cells in the gates are shown.

(D) The ratio of tdTomato-positive cells detected with flow cytometry. The results are shown in mean with SE (n = 4). * indicates the p value less than 0.05 calculated from Dunnett's test.

(E and F) Image of electrophoresis of genotyping PCR products. The results from 10 clones of EEF1-TEZ iPSCs are shown. The bands indicate KI alleles detected with chkEEF1A1 LAf and RFPr in (E) and WT detected with chkEEF1A1 LAf and chkEEF1A1 LAr in (F).

of zeocin. Surviving cells were re-seeded to 24-well plates with GCV at a concentration of 30 μg/mL for 2 days and then cultured without GCV. The medium was changed every other day, and replating was performed on days 10 and 16 (Figure 3A). After transfection and selection with zeocin only, around 3% of the cells were tdTomato positive (Figures 3B–3D). The data showed that the knockin of the *EEF1A1* gene was inefficient, resulting in most cells with randomly inserted donor vectors. After treatment with GCV, more than 10% of the cells were tdTomato detected with flow cytometry analysis and fluorescent imaging. We picked

up 10 clones and verified that all the clones carried knockin alleles without any detectable mutations (Figures 3E, 3F, and S2). These results suggested that tdTomato knocked-in cells were successfully enriched in the presence of GCV.

Efficient selection of H2B-EGFP and H2B-tdTomato knocked-in cells

H2BC21 encodes a replication-dependent histone that is a member of the histone H2B family. Because histone H2B is a subunit of the nucleosome complex, fluorescent reporter proteins fused to histone H2B are widely used as an indicator of chromosomal behavior and the cell cycle.[24,25] We constructed donor vectors for the C terminus of the *H2BC21* gene fused with the LG3 linker sequence and EGFP or tdTomato and bleomycin (zeocin)-resistance gene for knockin to assess the efficiency of GCV selection (Figure 4A). The hiPSC line 1383D6 was transfected with the double-tk donor vectors and the Cas9 nuclease/gRNA expression vector and selected in the presence of zeocin. Surviving cells (H2B-GEZ and H2B-TEZ) were transferred to 24-well plates at a density of 2.6 × 10^3 cells/cm^2. Only a small percentage of cells were fluorescence positive at this point (Figures 4B and 4C). Immediately after re-seeding, the cells were incubated with GCV at a concentration of 30 μg/mL for 2 days and then cultured without GCV. Most cells exhibited fluorescence (Figure 4C). GCV treatment resulted in a significantly higher ratio of tdTomato-positive cells detected with flow cytometry (Figures 4D and 4E). Then, 12 clones of fluorescent proteins from each condition were isolated and expanded from GCV-treated hiPSCs. The genotyping results showed that all clones carried knockin alleles (Figures 4F and 4G) without any detectable mutations (Figure S3). Two clones were homozygous for the insertion, while 22 clones were heterozygous. These results indicated that all established hiPSC clones carried the knockin allele. These knockin hiPSCs were positive for self-renewal marker proteins (Figures S4A and S4B). Upon differentiation, they contained three germ-layer-derived cells (Figures S4C and S4D). These results indicate that these knockin hiPSCs maintained pluripotency and self-renewal ability.

Efficient isolation of ISL1-tdTomato knocked-in cells after GCV selection

To assess whether double-tk donor vectors and GCV selection are applicable to knocking in to genes that expressed only after differentiation, but not in undifferentiated hiPSCs, we attempted to knock in tdTomato to the *ISL1* gene. *ISL1* encodes a transcription factor expressed in motor neurons, pancreatic progenitors, and a subset of cardiac progenitors.[26–28] The hiPSC line 1383D6 was transfected with the double-tk donor vector and the Cas9/gRNA expression vector. The resistant cells were selected in the presence of zeocin. The surviving cells (ISL1-TEZ) were transferred to 24-well plates, exposed to 30 μg/mL GCV for 2 days, and cultured in AK02N medium without GCV. Quantitative real-time PCR was used to investigate the knockin efficiency. We prepared two sets of primers, which could amplify only the knocked-in allele and the total (both knocked-in and wild-type) alleles, respectively (Figure 5A). Genomic DNA extracted from GCV-treated and untreated cells was serially diluted and subjected to quantitative PCR. The difference between the Ct values of the knockin and total alleles at each dilution point was determined, and their mean values were used to calculate the percentage of knockin alleles. We found that GCV-treated ISL1-TEZ cells contained 39% knockin alleles, whereas untreated ISL1-TEZ cells contained 1.4% knockin alleles. In the case of homozygous and heterozygous knockin, treatment with GCV concentrated 39%–68% ISL1-TEZ knockin cells compared to 1.4%–2.8% ISL1-TEZ knockin untreated cells (Figures 5B and 5C). Then, 23 clones were isolated and expanded from GCV-treated ISL1-TEZ hiPSCs. The result of genotyping of the right and left arms showed 16 out of 23 clones carrying knockin alleles (Figures 5D and 5E). These results indicated that knockin was established in approximately 70% of the cell population, which was consistent with the results of the quantitative genotyping shown above. Sequence data showed that five of the 26 alleles were intact after genome editing (Figure 5F).

To determine whether the obtained clones have differentiation potential, we tried to differentiate four randomly selected ISL1-TEZ hiPSC clones into motor neurons. Neural progenitor cells were induced with a medium supplemented with SB431542, DMH1, and CHIR99021, i.e., inhibitors of the transforming growth factor β (TGF-β)/Smad, BMP/Smad, and GSK3β/Wnt signaling pathways, respectively, for 6 days. For differentiation into motor neurons, these cells were further cultured in a medium containing retinoic acid, purmorphamine, and SAG dihydrochloride for 5 days. From day 11, the cells were cultured for 10 days in a medium containing the former plus brain-derived neurotrophic factor (BDNF). All four clones showed red fluorescence associated with the typical morphology of motor neurons (Figure 5G). These data indicate that GCV treatment of ISL1-tdTomato knocked-in cells facilitates the generation of hiPSC clones of interest without losing the ability for differentiation.

Efficient isolation of MYH7-tdTomato knocked-in cells after GCV selection

Finally, we attempted to knock in tdTomato to the *MYH7* gene, which is a cardiomyocyte marker gene.[29] The hiPSC line 1383D6 was transfected with the double-tk donor vector and Cas9/gRNA expression vector. The resistant cells were selected in the presence of zeocin. The surviving cells (MYH7-TEZ) were transferred to 24-well plates, treated with or without GCV for 2 days, and cultured in AK02N medium without GCV. Then, 16 clones from GCV-treated condition and 16 clones from non-treated conditions were isolated and examined. The result of genotyping PCR on the knockin allele showed that GCV-treated conditions contained a significantly higher ratio of knocked-in clones (Figures 6A–6C). We confirmed the self-renewal (Figures 6D and 6E) and pluripotency (Figure 6F) of these clones. We detected tdTomato fluorescence when these cells were differentiated into cardiomyocytes using a conventional induction method[30] (Figure 6G). These data indicate that GCV treatment of MYH7-tdTomato knocked-in cells facilitates the generation of hiPSC clones of interest without losing the ability for differentiation.

DISCUSSION

Although recent advances in genome editing technology with HDR using CRISPR-Cas9 have enabled the insertion of various

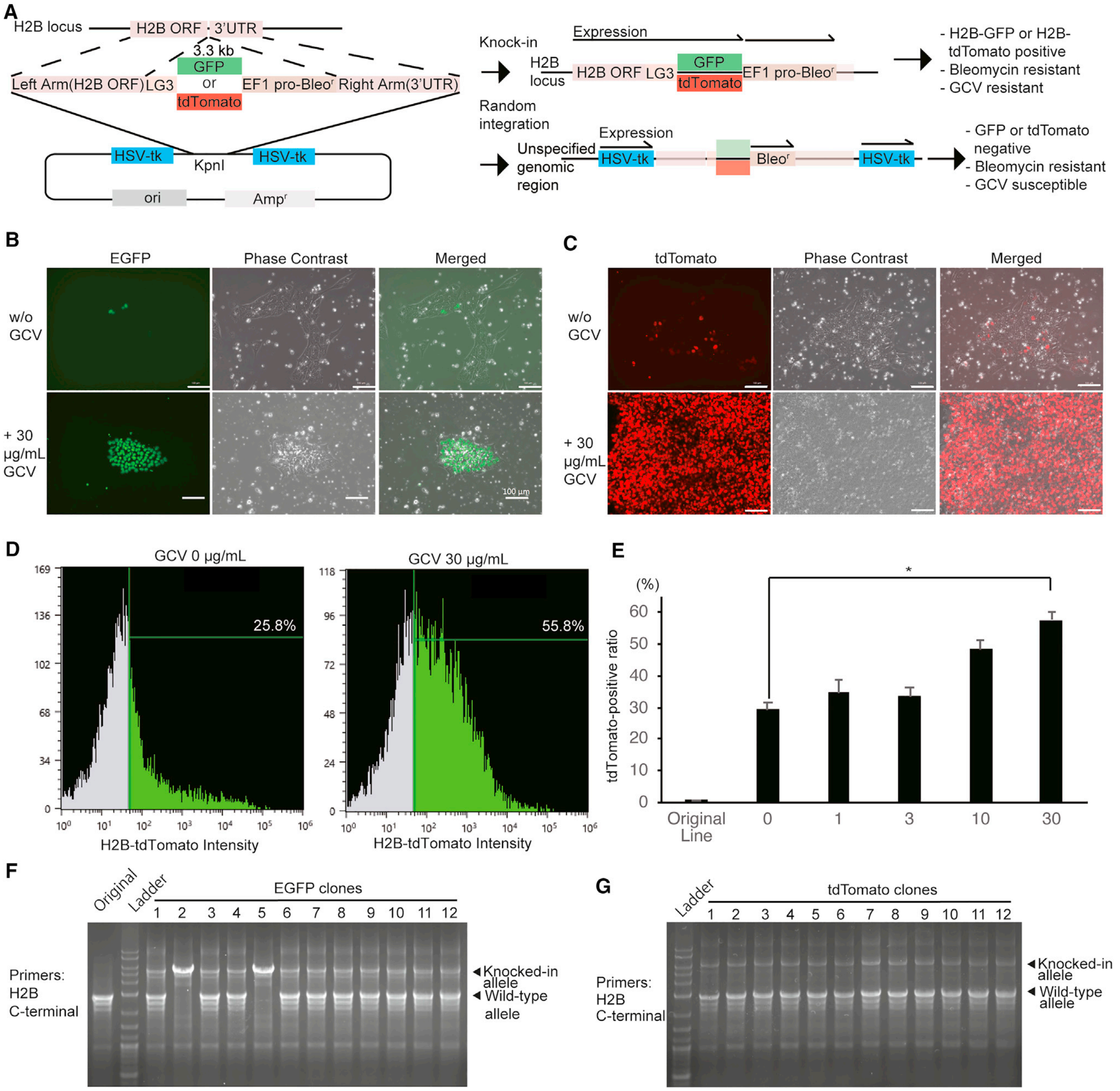

Figure 4. Selection of H2B-EGFP and H2B-tdTomato knocked-in cells by GCV/double-tk system

(A) The experimental scheme for the GCV selection of H2B-EGFP and H2B-tdTomato knocked-in hiPSCs.

(B and C) Microscope images of H2B-EGFP (B) and H2B-tdTomato (C) hiPSCs selected with 30 µg/mL GCV. Fluorescent and phase-contrast views of the cells after 20 days of GCV addition are shown. Scale bars, 100 µm.

(D) Representative images of flow cytometric analysis on EEF1A1-TEZ iPSCs selected with GCV at concentrations of 0 or 30 µg/mL. The percentages of the cells in the gates are shown.

(E) The ratio of tdTomato-positive cells detected with flow cytometry. The results are shown in mean with SE (n = 4). * indicates the p value less than 0.05 calculated from Dunnett's test.

(F and G) Genotyping PCR of hiPSC clones obtained from GCV-treated H2B-EGFP (F) and H2B-tdTomato (G). The primer set used in this genotyping PCR is a forward primer on the H2B C terminus and a reverse primer on each fluorescent protein gene.

reporter genes into the genome of mammalian cells, the efficiency is still low due to the random insertion of plasmid vectors carrying homology arms in unexpected sites. In this study, we developed the "double-tk donor vector system", in which the expression units of HSV-tk are placed on both outer sides of homology arms. We designed this system to efficiently eliminate

CellPress
OPEN ACCESS

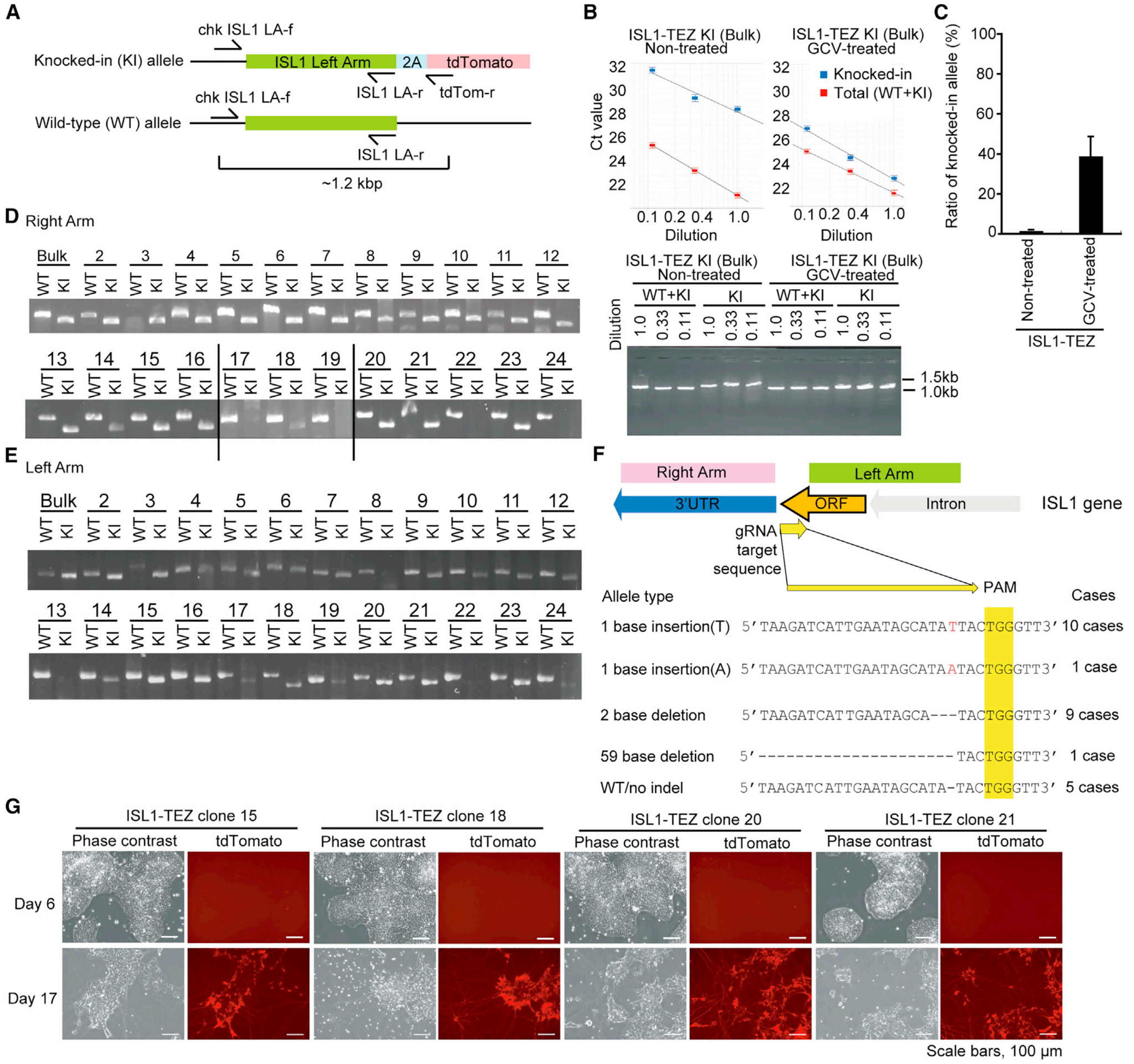

Figure 5. Generation of ISL1-tdTomato knocked-in cells after GCV selection

(A) Structure of the ISL1 gene near the end of the open reading frame and the primer binding sites for genotyping. The chk*ISL1* LA-f and tdTomato (tdTom)-r primer sets amplify only the left arm of the knockin (KI) allele, while the chk*ISL1* LA-f and *ISL1* LA-r sets can amplify both the KI and wild-type (WT) alleles. The sets of chk*ISL1* LA-f and *ISL1* RA-r3, chk*ISL1* RA-r and *ISL1* LA-f3, and chk*ISL1* RA-r and BleoR-f are for amplification of the WT left arm, WT right arm, and knocked-in right arm, respectively.

(B) Comparison of the probability of KI cells with or without GCV treatment. The genomic DNA extracted from GCV-treated and non-treated ISL1-TEZ cells were diluted by a factor of 3 and subjected to real-time PCR. The Ct values of DNA amplified from KI (blue square) and KI + WT (red square) alleles were plotted against relative DNA amount.

(C) Quantification of KI allele frequency calculated from real-time PCR. The results are shown as mean with SE (n = 3).

(D and E) Genotyping of ISL1 WT and KI alleles in bulk hiPSC population and 23 clones obtained from GCV-treated ISL1-TEZ cells.

(D) PCR for right arms with sets of primers, chkISL1 RA-r and ISL1 LA-f3 (for WT allele) or BleoR-f (for KI allele).

(E) PCR for left arms with sets of primers, chkISL1 LA-f and ISL1 RA-r3 (for WT allele) or tdTom-r (for KI allele).

(F) Sequence analysis for genotyping the right arm of ISL1 WT and KI alleles in hiPSC clones obtained from GCV-treated ISL1-TEZ cells.

(G) Differentiation of ISL1-TEZ iPSC clones into motor neurons. Fluorescent microscopic images of four randomly selected ISL1-EZ cell clones differentiated into motor neurons. Scale bar, 100 μm.

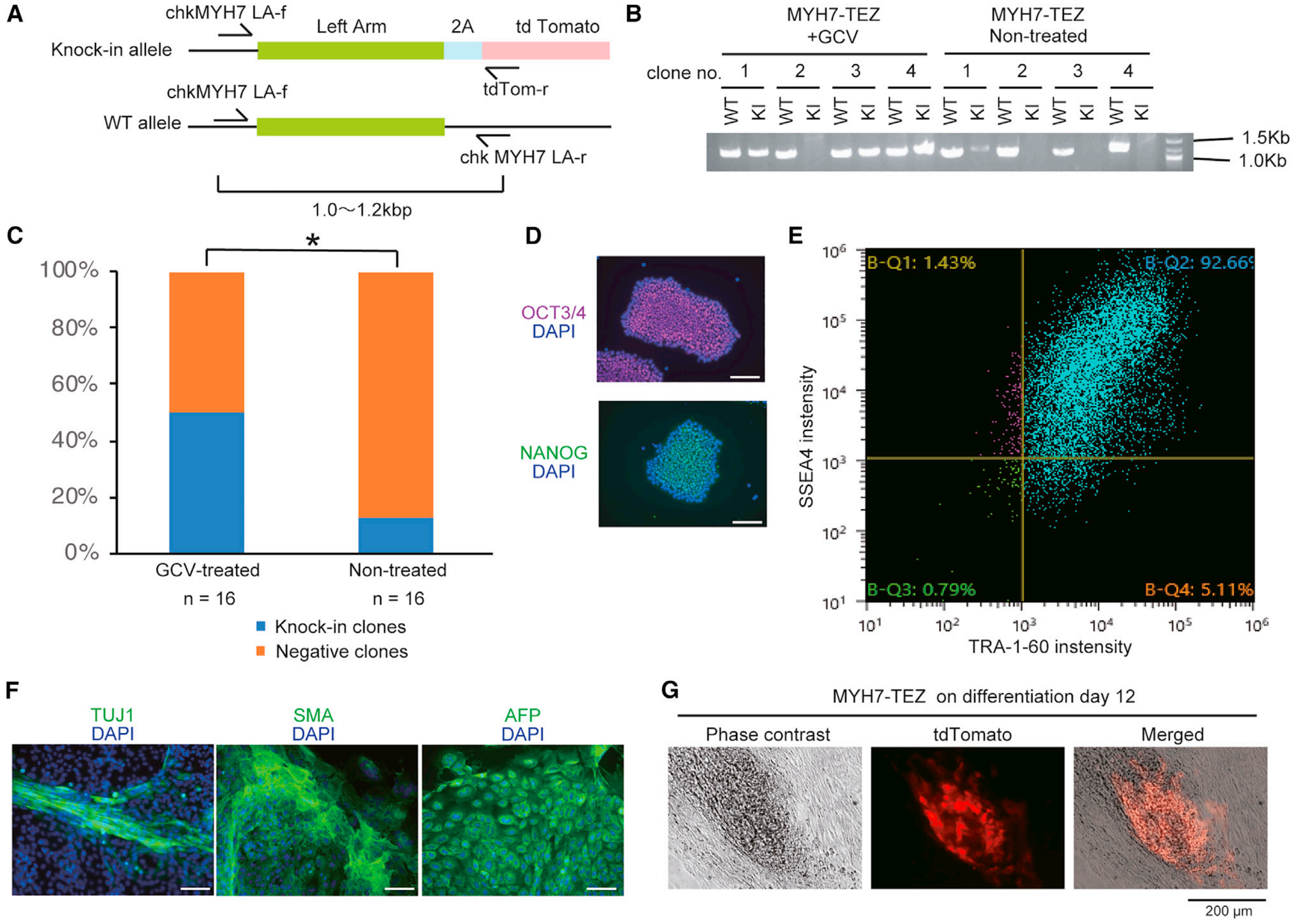

Figure 6. Generation of MYH7-tdTom knocked-in cells after GCV selection

(A) Structure of the MYH7 gene near the end of the open reading frame and the sites of the primer binding for genotyping. The chkMYH7 LA-f and tdTom-r primer sets amplify only the left arm of the KI allele, while the chk*MYH7* LA-f and *MYH71* LA-r sets can amplify the WT allele (KI allele could not be amplified with this primer set).

(B) Representative image of electrophoresis using the product of genotyping PCR for MYH7 WT and KI alleles in hiPSC clones obtained from GCV-treated or non-treated MYH7-TEZ cells.

(C) The ratio of KI clones and negative clones in GCV-treated or non-treated conditions. 16 clones from each condition were examined with genotyping PCR. *p <0.05 calculated from Fisher's exact test.

(D) Expression of self-renewal markers of hiPSCs, OCT3/4 (red) and NANOG (green), in a MYH7-TEZ clone. DAPI was used to stain nuclei (blue). Scale bars, 100 μm.

(E) Flow cytometry of TRA-1-60 and SSEA4 in a MYH7-TEZ clone.

(F) Pluripotency in a MYH7-TEZ clone was evaluated with EB formation assay. Immunocytochemistry of TUJ1 (ectoderm marker), alpha-SMA (mesoderm marker), and AFP (endoderm marker) in EB samples are shown in green. DAPI was used to stain nuclei (blue). Scale bars, 100 μm.

(G) Phase-contrast and tdTom fluorescent images in a MYH7-TEZ clone after being differentiated into cardiomyocytes on differentiation day 12. Scale bars, 200 μm.

unnecessary DNA fragments of the donor vectors inserted into the host genome or remaining in the cytoplasm. Using this system, we showed that knocked-in hiPSCs without random insertion were efficiently enriched in the presence of GCV. The effect of this negative selection using the double-tk donor vector system on the enrichment of knocked-in cells was confirmed using different target genes (i.e., *OCT4*, *EEF1A1*, *H2B*, *ISL1*, and *MYH7*), different gRNA sequences, different linkers (i.e., E2A peptide for separation and LG3 peptide for fusing), and different hiPSC lines (i.e., 454E2 and 1383D6). The efficiency of knocking in to a specific position in the genome of a cell is highly variable and unpredictable. This effect was particularly enhanced when the knockin efficiency was low, as we observed that the percentage of tdTomato-positive cells of the reporter at the *EEF1A1* locus increased from 1.4% to 69%. This increase enabled us to obtain knockin hiPSC clones effortlessly.

Visualizing the cellular state of differentiation in living cells facilitates studies on developmental biology and regenerative medicine. Our goal was to establish a simple and effective method to generate hiPSCs in which fluorescent proteins are knocked-in into desired marker genes. However, obtaining knocked-in cells targeting differentiated marker genes, which

are not expressed in undifferentiated hiPSCs, was technically demanding because discriminating knocked-in cells from randomly integrated cells required PCR genotyping. Our results showed that the GCV/double-tk system improved the efficiency of obtaining desired knockin strains by increasing their population. We used the example of the *ISL1* and *MYH7* genes, which are expressed only in specific differentiated cells but not in undifferentiated hiPSCs. These selection procedures did not impair their differentiation potentials, as it is evident that four randomly selected ISL1-TEZ clones treated with GCV were able to differentiate into motor neurons with increasing expression of the tdTomato reporter and typical neuron-like morphologies with neurite growth. The expression of ISL1 protein and tdTomato was consistent in differentiated motor neurons, and EB formation assays confirmed that these cells maintained their pluripotency.[31]

Limitations of study

In this study, we propose that the GCV/double-tk system helps obtain precise knocked-in cells. Although we have demonstrated the usefulness of this system only in hiPSCs, it could work in other cell types. Although a cloning process such as colony pickup is ultimately required to obtain knocked-in clones, GCV treatment facilitates the acquisition of target clones by increasing their population. This process is not always necessary when the knockin efficiency is high; however, it is advisable to construct a donor for insertion into a universal vector harboring elements that allow negative selection to obtain the desired knockin clone easily. Notably, because the sensitivity to GCV varies from cell to cell, the optimum conditions should be determined by titration experiments. We selected the cells at a lower concentration, as the cells were wiped out in most cases at a GCV concentration of 35 μg/mL or higher. When the donor vector is cleaved at more than one site and inserted into the genome in a form that does not contain HSV-tk, the cells cannot be eliminated by GCV treatment. In these cases, when we need to be careful about such microinsertions, we still need to validate authentic knockin cells by detailed PCR genotyping or massive sequencing. For future perspectives, this GCV/double-tk system can be combined with enhanced HDR methods using chemicals and other genetic manipulations.

STAR★METHODS

Detailed methods are provided in the online version of this paper and include the following:

- KEY RESOURCES TABLE
- RESOURCE AVAILABILITY
  - Lead contact
  - Materials availability
  - Data and code availability
- EXPERIMENTAL MODEL AND STUDY PARTICIPANT DETAILS
  - hiPSC maintenance culture
- METHOD DETAILS
  - CRISPR-Cas9 vector construction
  - Gene transfection
  - GCV selection
  - Three-germ-layer differentiation
  - Immunocytochemistry
  - Flow cytometry
  - Genotyping PCR
  - Quantitative PCR-based genotyping
  - Sequencing
  - Motor neuron differentiation
  - Cardiomyocyte differentiation
- QUANTIFICATION AND STATISTICAL ANALYSIS

SUPPLEMENTAL INFORMATION

Supplemental information can be found online at https://doi.org/10.1016/j.crmeth.2023.100662.

ACKNOWLEDGMENTS

We express our sincere gratitude to Ms. Kumiko Omori for her administrative support. This research was supported in part by grants from AMED (23bm1423010h0001) to Y. Hayashi; AMED (21ae0201004s0504) to Y.M.; JST (JPMJPF2017) to Y.M.; JSPS KAKENHI Grant-in-Aid for Young Scientists (A) (17H05063) to Y. Hayashi; JSPS KAKENHI Grant-in-Aid for Scientific Research (C) (21K05994) to Y.M.; and the Uehara Memorial Foundation.

AUTHOR CONTRIBUTIONS

Conceptualization, K. Nakade and Y. Hayashi; methodology, K. Nakade, S.T., K. Nakashima, Y.A., I.S., J.L., Y. Hemmi, and Y. Hayashi; validation, K. Nakade, S.T., K. Nakashima, Y.A., I.S., J.L., Y. Hemmi, Y.S., Y.M., and Y. Hayashi; formal analysis, K. Nakade, S.T., and Y. Hayashi; investigation, K. Nakade, S.T., K. Nakashima, Y.A., I.S., J.L., Y. Hemmi, Y.S., and Y. Hayashi; resources, K. Nakade, Y.M., and Y. Hayashi; writing – original draft, K. Nakade and Y. Hayashi; writing – review & editing, all authors; visualization, K. Nakade, S.T., K. Nakashima, Y.A., I.S., J.L., Y. Hemmi, Y.S., and Y. Hayashi; supervision, K. Nakade, Y.M., and Y. Hayashi; project administration, K. Nakade and Y. Hayashi; funding acquisition, K. Nakade, Y.M., and Y. Hayashi.

DECLARATION OF INTERESTS

The authors declare no competing interests.

Received: November 22, 2022
Revised: October 2, 2023
Accepted: November 15, 2023
Published: December 11, 2023

REFERENCES

1. Thomson, J.A., Itskovitz-Eldor, J., Shapiro, S.S., Waknitz, M.A., Swiergiel, J.J., Marshall, V.S., and Jones, J.M. (1998). Embryonic stem cell lines derived from human blastocysts. Science *282*, 1145–1147.
2. Takahashi, K., Tanabe, K., Ohnuki, M., Narita, M., Ichisaka, T., Tomoda, K., and Yamanaka, S. (2007). Induction of pluripotent stem cells from adult human fibroblasts by defined factors. Cell *131*, 861–872.
3. Yu, J., Vodyanik, M.A., Smuga-Otto, K., Antosiewicz-Bourget, J., Frane, J.L., Tian, S., Nie, J., Jonsdottir, G.A., Ruotti, V., Stewart, R., et al. (2007). Induced pluripotent stem cell lines derived from human somatic cells. Science *318*, 1917–1920.
4. Deltcheva, E., Chylinski, K., Sharma, C.M., Gonzales, K., Chao, Y., Pirzada, Z.A., Eckert, M.R., Vogel, J., and Charpentier, E. (2011). CRISPR RNA maturation by trans-encoded small RNA and host factor RNase III. Nature *471*, 602–607.

5. Mali, P., Yang, L., Esvelt, K.M., Aach, J., Guell, M., DiCarlo, J.E., Norville, J.E., and Church, G.M. (2013). RNA-guided human genome engineering via Cas9. Science *339*, 823–826.
6. Hou, Z., Zhang, Y., Propson, N.E., Howden, S.E., Chu, L.F., Sontheimer, E.J., and Thomson, J.A. (2013). Efficient genome engineering in human pluripotent stem cells using Cas9 from Neisseria meningitidis. Proc. Natl. Acad. Sci. USA *110*, 15644–15649.
7. Zhang, J.P., Li, X.L., Li, G.H., Chen, W., Arakaki, C., Botimer, G.D., Baylink, D., Zhang, L., Wen, W., Fu, Y.W., et al. (2017). Efficient precise knockin with a double cut HDR donor after CRISPR/Cas9-mediated double-stranded DNA cleavage. Genome Biol. *18*, 35.
8. Anzai, T., Hara, H., Chanthra, N., Sadahiro, T., Ieda, M., Hanazono, Y., and Uosaki, H. (2021). Generation of Efficient Knock-in Mouse and Human Pluripotent Stem Cells Using CRISPR-Cas9. Methods Mol. Biol. *2320*, 247–259.
9. Lin, S., Staahl, B.T., Alla, R.K., and Doudna, J.A. (2014). Enhanced homology-directed human genome engineering by controlled timing of CRISPR/Cas9 delivery. Elife *3*, e04766.
10. Maurissen, T.L., and Woltjen, K. (2020). Synergistic gene editing in human iPS cells via cell cycle and DNA repair modulation. Nat. Commun. *11*, 2876.
11. Arnoult, N., Correia, A., Ma, J., Merlo, A., Garcia-Gomez, S., Maric, M., Tognetti, M., Benner, C.W., Boulton, S.J., Saghatelian, A., and Karlseder, J. (2017). Regulation of DNA repair pathway choice in S and G2 phases by the NHEJ inhibitor CYREN. Nature *549*, 548–552.
12. Nambiar, T.S., Billon, P., Diedenhofen, G., Hayward, S.B., Taglialatela, A., Cai, K., Huang, J.W., Leuzzi, G., Cuella-Martin, R., Palacios, A., et al. (2019). Stimulation of CRISPR-mediated homology-directed repair by an engineered RAD18 variant. Nat. Commun. *10*, 3395.
13. Haapaniemi, E., Botla, S., Persson, J., Schmierer, B., and Taipale, J. (2018). CRISPR-Cas9 genome editing induces a p53-mediated DNA damage response. Nat. Med. *24*, 927–930.
14. Ihry, R.J., Worringer, K.A., Salick, M.R., Frias, E., Ho, D., Theriault, K., Kommineni, S., Chen, J., Sondey, M., Ye, C., et al. (2018). p53 inhibits CRISPR-Cas9 engineering in human pluripotent stem cells. Nat. Med. *24*, 939–946.
15. Hockemeyer, D., Soldner, F., Beard, C., Gao, Q., Mitalipova, M., DeKelver, R.C., Katibah, G.E., Amora, R., Boydston, E.A., Zeitler, B., et al. (2009). Efficient targeting of expressed and silent genes in human ESCs and iPSCs using zinc-finger nucleases. Nat. Biotechnol. *27*, 851–857.
16. Ashton, W.T., Karkas, J.D., Field, A.K., and Tolman, R.L. (1982). Activation by thymidine kinase and potent antiherpetic activity of 2’-nor-2’-deoxyguanosine (2’NDG). Biochem. Biophys. Res. Commun. *108*, 1716–1721.
17. Smith, K.O., Galloway, K.S., Kennell, W.L., Ogilvie, K.K., and Radatus, B.K. (1982). A new nucleoside analog, 9-[[2-hydroxy-1-(hydroxymethyl) ethoxyl]methyl]guanine, highly active in vitro against herpes simplex virus types 1 and 2. Antimicrob. Agents Chemother. *22*, 55–61.
18. Moolten, F.L. (1986). Tumor Chemosensitivity Conferred by Inserted Herpes Thymidine Kinase Genes - Paradigm for a Prospective Cancer Control Strategy. Cancer Res. *46*, 5276–5281.
19. Schwartz, F., Maeda, N., Smithies, O., Hickey, R., Edelmann, W., Skoultchi, A., and Kucherlapati, R. (1991). A Dominant Positive and Negative Selectable Gene for Use in Mammalian-Cells. P Natl Acad Sci USA *88*, 10416–10420.
20. Naujok, O., Kaldrack, J., Taivankhuu, T., Jörns, A., and Lenzen, S. (2010). Selective removal of undifferentiated embryonic stem cells from differentiation cultures through HSV1 thymidine kinase and ganciclovir treatment. Stem Cell Rev. Rep. *6*, 450–461.
21. Iwasawa, C., Tamura, R., Sugiura, Y., Suzuki, S., Kuzumaki, N., Narita, M., Suematsu, M., Nakamura, M., Yoshida, K., Toda, M., et al. (2019). Increased Cytotoxicity of Herpes Simplex Virus Thymidine Kinase Expression in Human Induced Pluripotent Stem Cells. Int. J. Mol. Sci. *20*, 810. ARTN 810.
22. Niwa, H., Miyazaki, J., and Smith, A.G. (2000). Quantitative expression of Oct-3/4 defines differentiation, dedifferentiation or self-renewal of ES cells. Nat. Genet. *24*, 372–376.
23. Lund, A., Knudsen, S.M., Vissing, H., Clark, B., and Tommerup, N. (1996). Assignment of human elongation factor 1 alpha genes: EEF1A maps to chromosome 6q14 and EEF1A2 to 20q13.3. Genomics *36*, 359–361.
24. Hadjantonakis, A.K., and Papaioannou, V.E. (2004). Dynamic in vivo imaging and cell tracking using a histone fluorescent protein fusion in mice. BMC Biotechnol. *4*, 33. Artn 33.
25. Fraser, S.T., Hadjantonakis, A.K., Sahr, K.E., Willey, S., Kelly, O.G., Jones, E.A.V., Dickinson, M.E., and Baron, M.H. (2005). Using a histone yellow fluorescent protein fusion for tagging and tracking endothelial cells in ES cells and mice. Genesis *42*, 162–171.
26. Karlsson, O., Thor, S., Norberg, T., Ohlsson, H., and Edlund, T. (1990). Insulin gene enhancer binding protein Isl-1 is a member of a novel class of proteins containing both a homeo- and a Cys-His domain. Nature *344*, 879–882.
27. Pfaff, S.L., Mendelsohn, M., Stewart, C.L., Edlund, T., and Jessell, T.M. (1996). Requirement for LIM homeobox gene Isl1 in motor neuron generation reveals a motor neuron-dependent step in interneuron differentiation. Cell *84*, 309–320.
28. Bu, L., Jiang, X., Martin-Puig, S., Caron, L., Zhu, S., Shao, Y., Roberts, D.J., Huang, P.L., Domian, I.J., and Chien, K.R. (2009). Human ISL1 heart progenitors generate diverse multipotent cardiovascular cell lineages. Nature *460*, 113–117.
29. Saez, L., and Leinwand, L.A. (1986). Characterization of diverse forms of myosin heavy chain expressed in adult human skeletal muscle. Nucleic Acids Res. *14*, 2951–2969.
30. Lian, X., Zhang, J., Azarin, S.M., Zhu, K., Hazeltine, L.B., Bao, X., Hsiao, C., Kamp, T.J., and Palecek, S.P. (2013). Directed cardiomyocyte differentiation from human pluripotent stem cells by modulating Wnt/beta-catenin signaling under fully defined conditions. Nat. Protoc. *8*, 162–175.
31. Tsukamoto, S., Nakade, K., Wakabayashi, T., Nakashima, K., Takami, M., Hemmi, Y., Kuramochi, Y., Shimizu, T., Arai, Y., Matsuo-Takasaki, M., et al. (2021). Generation of two ISL1-tdTomato reporter human induced pluripotent stem cell lines using CRISPR-Cas9 genome editing. Stem Cell Res. *53*, 102363. ARTN 102363.
32. Okita, K., Matsumura, Y., Sato, Y., Okada, A., Morizane, A., Okamoto, S., Hong, H., Nakagawa, M., Tanabe, K., Tezuka, K.i., et al. (2011). A more efficient method to generate integration-free human iPS cells. Nat. Methods *8*, 409–412.
33. Nakagawa, M., Taniguchi, Y., Senda, S., Takizawa, N., Ichisaka, T., Asano, K., Morizane, A., Doi, D., Takahashi, J., Nishizawa, M., et al. (2014). A novel efficient feeder-free culture system for the derivation of human induced pluripotent stem cells. Sci. Rep. *4*, 3594.

CellPress
OPEN ACCESS

STAR★METHODS

KEY RESOURCES TABLE

REAGENT or RESOURCE	SOURCE	IDENTIFIER
Antibodies		
Mouse anti-OCT3/4 (1:500)	Santa Cruz Biotechnology	Cat# sc-5279; RRID:AB_628051
Rabbit anti-OCT3/4 (1:100)	Santa Cruz Biotechnology	Cat# sc-9081; RRID:AB_2167703
Rabbit anti-NANOG (1:500)	ReproCELL	Cat# RCAB004P-F; RRID:AB_1560380
APC anti-human SSEA-4 Antibody (1:100)	BioLegend	Cat# 330418; RRID:AB_2616819
Alexa Fluor 488 Mouse anti-TRA-1-60 (1:100)	BioLegend	Cat# 330613; RRID:AB_2295395
Mouse anti-TUJ1 (1:500)	R and D Systems	Cat# MAB1195; RRID:AB_357520
Mouse anti-SMA (1:500)	R and D Systems	Cat# MAB1420; RRID:AB_262054
Mouse anti-AFP (1:500)	R and D Systems,	Cat# MAB1368; RRID:AB_357658
Mouse anti-ISL1 (2 μg/mL))	DSHB	Cat# 40.2D6; RRID:AB_528315
Donkey anti-Mouse IgG Alexa Flour 488 (1:2,000)	Thermo Fisher Scientific	Cat# A-21202; RRID:AB_141607
Donkey anti-Mouse IgG (H + L) Highly Cross-Adsorbed Secondary Antibody, Alexa Fluor 555	Thermo Fisher Scientific	Cat# A-31570; RRID:AB_2536180
Donkey anti-rabbit IgG-FITC (1:100)	Santa Cruz Biotechnology	Cat# sc-2090; RRID:AB_641179
Chemicals, peptides, and recombinant proteins		
StemFit AK02N medium	Ajinomoto	AK02N
iMatrix-511 silk	Matrixome	892021
Y-27632	Wako	HY-10071
CTS TrypLE select	Thermo Fisher Scientific	A12859-01
Zeocin	Invivogen	ant-zn-1
Ganciclovir	Wako	078–04481
DMEM with high glucose	Nacalai Tesque	08458–16
DMEM with high glucose	Wako	197–16275
RPMI-1640 medium	Wako	189–02025
Penicillin/Streptomycin solution	Nacalai Tesque	26253–84
Fetal Bovine Serum	Biosera	1003/500
B27 supplement, minus insulin	Thermo Fisher Scientific	A1895601
0.1% (w/v) gelatin solution	Wako	190–15805
SB431542	Wako	312491
DMH1	Wako	041–33881
CHIR99021	Wako	034–23103
IWP-2	Wako	030–24303
All-trans retinoic acid	Wako	188–01113
SAG	Sigma Aldrich	566661
Purmorphamine	Wako	166–23991
BDNF	Wako	028–16451
Tks Gflex DNA polymerase	Takara	R060A

(Continued on next page)

Continued		
REAGENT or RESOURCE	SOURCE	IDENTIFIER
Midori Green Advance	Nippon Genetics	NE-MG04
SYBR Green	Thermo Fisher Scientific	S7563
Critical commercial assays		
In-Fusion HD Cloning Kit	Takara-Clontech	639648
Lipofectamine Stem Transfection Reagent	Thermo Fisher Scientific	STEM00003
BD Cytofix/Cytoperm kit	BD	554714
DNeasy Blood and Tissue Kit	QIAGEN	#69506
ExoSAP-IT^{TM} PCR Product Cleanup Reagent	Thermo Fisher Scientific	78201.1.ML
BigDye^{TM} Terminator v3.1 Cycle Sequencing Kit	Thermo Fisher Scientific	4337457
Experimental models: Cell lines		
Human iPSC line: 454E2	RIKEN Cell Bank	HPS0077; RRID:CVCL_T791
Human iPSC line: 1383D6	RIKEN Cell Bank	HPS1006; RRID:CVCL_UP39
Human fibroblast line: WI-38	RIKEN Cell Bank	RCB0702; RRID:CVCL_0579
Human iPSC line: OCT4-TEZ	RIKEN Cell Bank (generated in this study)	HPS5232
Human iPSC line: H2B-TEZ	RIKEN Cell Bank (generated in this study)	HPS5140
Human iPSC line: H2B-GEZ	RIKEN Cell Bank (generated in this study)	HPS5141
Human iPSC line: ISL1-TEZ_1	RIKEN Cell Bank (generated in this study)	HPS5037
Human iPSC line: ISL1-TEZ_2	RIKEN Cell Bank (generated in this study)	HPS5038
Oligonucleotides		
Primers for plasmid constructs, sequencing, and PCR, see Table S1	This paper	N/A
Recombinant DNA		
Plasmids for genome editing, see Table S2	Addgene or RIKEN DNA bank	N/A

RESOURCE AVAILABILITY

Lead contact

Further information and requests for resources and reagents should be directed to and will be fulfilled by the lead contact, Yohei Hayashi (yohei.hayashi@riken.jp).

Materials availability

Cell lines used in this study are available from RIKEN BRC Cell bank. Accession numbers are listed in the key resources table. Plasmids used and newly generated in this study are listed with accession numbers in Table S2. Newly generated plasmids were deposited to RIKEN DNA bank.

Data and code availability

- The datasets supporting the current study have not been deposited in a public repository but are available from the lead contact on request.
- This study does not report original code.
- Any additional information required to reanalyze the data reported in this paper is available from the lead contact upon request.

EXPERIMENTAL MODEL AND STUDY PARTICIPANT DETAILS

hiPSC maintenance culture

The hiPSC cells, 454E2 line (HPS0077, RIKEN BRC, female)[32] and 1383D6 line (HPS1006, RIKEN BRC, male),[33] were obtained from RIKEN BioResource Research Center (BRC), Cell Engineering Division (RIKEN cell bank). To culture hiPSCs under feeder-free conditions, StemFit AK02N medium (Ak02N, Ajinomoto, Japan) and polystyrene tissue culture plates coated with Laminin-511 fragment (iMatrix-511 silk; 892021, Matrixome, Japan) were used. On the first day after passage, AK02N medium supplemented with 10 μM Y-27632 (HY-10071; Wako, Japan) was used. The medium was changed every other day from the day after the passage. The iPSCs

were passaged every 6–8 days using 0.5 × CTS TrypLE Select (A12859-01, Thermo Fisher Scientific, USA) supplemented with 0.5 mM EDTA in PBS(−) or only 0.5 mM EDTA in PBS(−).

METHOD DETAILS

CRISPR-Cas9 vector construction

For Cas9 nuclease/gRNA expression vectors, the expression vectors for Cas9 Nuclease and the gRNA were constructed by inserting annealed synthetic oligomers into the BbsI site of pX330 (for cleavage of *OCT3/4*, *H2B*, or *ISL1*, #42230, Addgene) or pX459 (for cleavage of *EEF1A1*, #62988, Addgene). The sequences of the synthetic oligomers pX330-gOCT3/4 (RDB19580, RIKEN BRC), pX459-gEEF1A1 (RDB19570, RIKEN BRC), pX330-gH2B (RDB19579, RIKEN BRC), pX330-gISL1 (RDB18671, RIKEN BRC), and pX330-gMYH7 (RDB20121, RIKEN BRC) are shown in Table S1.

For donor vectors, universal vectors were designed to insert homology arms, including reporter genes, into the KpnI site. To construct donor vectors for *OCT3/4* and *EEF1A1* genes, DNA fragments around the stop codon from 1 kbp upstream to 1 kbp downstream were amplified from the genomic DNA of WI38 cells (RCB0702, RIKEN BRC) by PCR using a set of primers, OCT3/4 LA-f and OCT3/4 RA-r for *OCT3/4*, EEF1A1 LA-f and EEF1A1 RA-r for *EEF1A1*, and MYH7 LA-f and MYH7 RA-r for MYH7. The PCR product was cloned into the KpnI site of pUCtk2×2 [RDB19569, RIKEN BRC, a derivative of pCRtk2×2NN (RDB18670, RIKEN BRC), in which the $Bleo^R$ gene was replaced with Amp^R] to generate pUCtk2×2-*OCT3/4* and *EEF1A1* using an In-Fusion HD Cloning Kit (639648, Takara-Clontech). Each pUCtk2×2- series vector containing these inserts was amplified into two fragments by PCR using the following sets of primers: OCT3/4 LAr and p15AoriF and OCT3/4 RA-f and p15AoriR for *OCT3/4*, EEF1A1 LAr and p15AoriF and EEF1A1 RA-f and p15AoriR for *EEF1A1*. To introduce the reporter-selection marker, a part of the pUC-TEZ vector (RDB18672, RIKEN BRC) harboring 2A-tdTomato and EF1 alpha promoter-driven bleomycin-resistance gene was amplified using a set of primers, 2Af-1-16 and Bleo-R inf-puro. The PCR fragments were subjected to in-fusion cloning to generate pUCtk2×2-OCT3/4-TEZ (RDB19572, RIKEN BRC) pUCtk2×2-EEF1A1-TEZ (RDB19571, RIKEN BRC), and pUCtk2x2-MYH7-TEZ (RDB20120, RIKEN BRC).

To construct the donor vector for H2BC21, the left- and right-HDR arms were amplified by PCR using genomic DNA from WI38 cells as a template with primer sets H2B LA-f and H2B LA-r and H2B RA-f and H2B RA-r, respectively. Subsequently, fragments of the left and right arms were assembled and re-amplified using the H2B LA-f and H2B RA-r primer set. The resulting DNA fragment was cloned into the *Kpn* I site of pUCtk2×2-UAS (RDB19575, RIKEN BRC), a pUCtk2×2 derivative having an upstream activation sequence (UAS), using an In-Fusion HD Cloning Kit to generate pUCtk2X2-H2B (RDB19576, RIKEN BRC). To prepare an enhanced green fluorescent protein (EGFP) reporter fragment (2xLG3-GEZ), a 2xLG3 fragment (synthetic peptide sequence based on the scFv of immunoglobulins, described in JP2009261259A) was amplified by PCR from pUC-2xLG3-TEZ (RDB19574, RIKEN BRC), a vector in which the 2xLG3 linker was replaced by the 2A peptide of pUC-TEZ, with the set of primers M13RV and tdTom-r. The GFP fragment was amplified from pEGFP-N1 (#6085-1, Clontech) by PCR using the set of primers GFP-f and GFP-r. The former and latter DNA fragments were combined and re-amplified utilizing a set of primers, M13RV and GFP-r. The generated DNA fragment was digested with *Hin*dIII/*Bsr*GI and inserted into the *Hin*dIII/*Bsr*GI site of pUC-2xLG3-TEZ to generate pUC-2xLG3-GEZ (RDB19573; RIKEN BRC) by ligation. Finally, the fluorescent reporter fragments 2xLG3-GEZ and 2xLG3-TEZ released by *Eco* RV digestion were inserted into the *Kpn* I site between the left and right arms of pUCtk2X2-H2B using an In-Fusion HD cloning kit to establish pUC2×2Tk-H2B-GEZ (RDB19577, RIKEN BRC) and pUC2×2Tk-H2B-TEZ (RDB19578, RIKEN BRC), respectively. Construction of the donor vector for the *ISL1* gene (pCRtk2×2-ISL1-TEZ, RDB 18669, RIKEN BRC) has been previously described.[31] The sequences of the primer sets used in this study are summarized in Table S1. All the DNA plasmids used in this study are listed in Table S2.

Gene transfection

For generating knocked-in cells, we introduced 1.5 μg of donor vectors [pUC-OCT3/4-TEZ (RDB 19586, RIKEN BRC), pUCtk2x2-OCT3/4-TEZ, pUCtk2x2-EEF1A1-TEZ or pUCtk2x2-MYH7-TEZ] and 0.5 μg of Cas9-gRNA expression vector (px330-gOCT3/4, px459-gEEF1, or px330-gMYH7) into 2 x 10^4 cells of hiPSC 454E2 (for OCT3/4) or 1383D6 (for EEF1A1) lines using Lipofectamine Stem Transfection Reagent (STEM00003, Thermo Fisher Scientific) following the manufacturer's instruction. Alternatively, we introduced 5 μg of donor vector (pCRtk2×2-ISL1-TEZ, pUC2×2Tk-H2B-GEZ, or pUC2×2Tk-H2B-TEZ) and 1 μg of Cas9-gRNA expression vector (pX330-gISL1 or pX330-gH2B) into the 1383D6 hiPSC line using an electroporator (NEPA21, Nepagene). Four days after transfection, the cells were selected in the presence of zeocin (2 μg/mL; ant-zn-1, Invivogen). All the plasmids used in this study are listed in Table S2.

GCV selection

After transfection and the zeocin selection, survived hiPSCs were seeded in a 24-well plate at a density of 5 × 10^3 cells/well. Ganciclovir (GCV; 078–04481, Wako) was added at the indicated concentrations immediately after seeding. The cells were cultured for one or two days, and the medium was replaced with fresh medium without GCV. The medium was changed once a day for an additional 2 days, and the selected cells proliferated without GCV. The cells were passaged on days around 10 and 16 after GCV treatment. These selected cells were expanded to collect frozen stocks or seeded at 100 cells/cm^2 to be isolated as single cell-derived colonies.

Three-germ-layer differentiation

Embryoid body (EB) formation assay was performed for three-germ-layer differentiation. Briefly, knock-in hiPSCs were cultured in DMEM high Glucose (197–16275, Wako) supplemented with 10% (v/v) fetal bovine serum (FBS 1003/500, Biosera) (EB medium) on a low-attachment dish for 8 days to form EBs. Then these cell aggregates were transferred to a tissue culture dish coated with gelatin (pre-coated with 0.1% (w/v) gelatin solution, 190–15805, Wako) for another 8 days to be examined with fluorescence imaging and flow cytometry. Differentiation was validated by immunocytochemistry, as described below.

Immunocytochemistry

Immunocytochemistry was performed to detect the expression of self-renewal (OCT4 and NANOG) and pluripotency markers (TUJ1, SMA, and AFP). Briefly, cells were fixed with PBS containing 4% paraformaldehyde for 10 min at room temperature, permeabilized in PBS containing 0.1% Triton X-100 for another 10 min at room temperature, and then washed with PBS. Primary antibodies were incubated with 0.1% FBS in PBS overnight at 4°C. The secondary antibodies were incubated for 1 h at room temperature in PBS with 0.1% bovine serum albumin (BSA). The antibodies used in this study are listed in the key resources table. Cell nuclei were stained with the Fluoro-KEEPER Antifade Reagent DAPI (12745-74, Nacalai Tesque). Images were taken using an all-in-one fluorescence microscope (BZ-X800; KEYENCE).

Flow cytometry

The cells were detached using 0.5 × TrypLE Select CTS supplemented with 0.5 mM EDTA in PBS(−). These dissociated cells were subjected to FACS analysis either directly or after immunofluorescence staining using the Guava easyCyte HT System (6HT2L, Millipore). For immunofluorescence staining, the cells were fixed and permeabilized using a BD Cytofix/Cytoperm kit (554714, BD Bioscience) and stained with rabbit anti-OCT3/4 antibody and donkey anti-rabbit IgG-FITC as the primary and secondary antibody, respectively, at 1/1000 dilution for 1 h. The antibodies used in this study are listed in the key resources table.

Genotyping PCR

Prior to genotyping, antibiotic-resistant cells were expanded to 100 cells/cm^2 to isolate single-cell-derived colonies. The isolated clones were examined for the presence of targeted insertions by PCR. Genomic DNA was extracted from hiPSCs using a DNeasy Blood and Tissue Kit (#69506, QIAGEN). Genotyping PCR analysis was performed using Tks Gflex DNA polymerase (R060A; Takara) following the manufacturer's instructions. The primer sets used for genotyping were *H2BC21*_genotype F and R (for *H2B*), hsv-tk_genotype F and R (for HSV-tk), tdTom-r or *ISL1* RA-r3 and chkISL1 LA-f (for *ISL1* left arm), and BleoR-f or *ISL1* LA-f3 and chk*ISL1* RA-r (for *ISL1* right arm).

Electrophoresis was performed on the PCR products on a 1% agarose gel. These gel samples were stained with Midori Green Advance (NE-MG04; Nippon Genetics) and visualized using a Blue LED in Printgraph 2M (WSE-5200; ATTO). The sequences of the primer sets used in this study are summarized in Table S1.

Quantitative PCR-based genotyping

Knocked-in cells were lysed in PBS(−) supplemented with 1% SDS. The cell lysate was extracted with phenol/chloroform, and genomic DNA was precipitated with ethanol and resuspended in Tris-EDTA buffer. For quantitative genotyping, Tks Gflex DNA Polymerase (R060A, Takara-Clontech) supplemented with 1 x SYBR Green (S7563, Thermo Fisher Scientific) and 0.25 μM of specific primers for wild-type and knocked-in allele. The sets of primers for detecting both wild-type and knocked-in alleles and only the knocked-in alleles were chkISL1 LA-f and ISL1 LA-r and chkISL1 LA-f and tdTom-r, respectively. Quantitative PCR was performed on a StepOne Plus real-time thermal cycler (Thermo Fisher Scientific), and the Ct values of each sample containing diluted genomic DNA were measured. The sequences of the primers used in this study are summarized in Table S1.

Sequencing

For sequencing, PCR products from genotyping were treated with ExoSAP-IT PCR Product Cleanup Reagent (78201.1.ML, Applied Biosystems) and used as a template for the reaction. The sequencing reaction was performed using specific primers, the BigDye Terminator v3.1 Cycle Sequencing Kit (4337457, Applied Biosystems), and a 3500 Genetic Analyzer (4440462, Applied Biosystems) according to the manufacturer's instructions. Sequences of primers used in this study are summarized in Table S1.

Motor neuron differentiation

hiPSCs were differentiated into motor neurons as we reported previously.[31] Briefly, hiPSCs were seeded at 2,500 cells/cm^2 on iMatrix-coated dish with Y-27632. On the following day (as Day 0), the medium was replaced with Differentiation medium1; DM1, which is consisted of StemFit AK02N without supplement C, 1% Penicillin/Streptomycin solution (P/S) (26253-84, Nacalai Tesque) containing 10 μM SB431542 (312491, Wako), 10 μM DMH1 (041–33881, Wako) and 3 μM CHIR99021 (034–23103, Wako). The culture medium was changed every other day. iPSCs were cultured in DM1 for 6 days to induce neural progenitor cells. On Day 6, for motor neuron induction from neural progenitor cells, DM1 was replaced with DM2, which consists of StemFit AK02N without supplement C, 1% P/S containing 1 μM of all-trans retinoic acid (188–01113, Wako), SAG (566661, Sigma Aldrich) and Purmorphamine (166–23991, Wako). On day 10, the neural progenitor cells were dissociated with 0.5 mM EDTA in PBS and seeded at 2,000 cells/cm^2 on iMatrix

511-coated plate in DM2 with Y-27632. On Day 11, DM2 medium was replaced to DM2 with 10 ng/mL BDNF (028–16451, Wako). The medium was changed every other day until analysis.

Cardiomyocyte differentiation

hiPSCs were differentiated into cardiomyocytes as a previous study.[30] Briefly, four days before induction, the cells were plated at 2×10^4 cells/cm^2 in Stem Fit AK02N medium with 10 μM Y-27632. After 3–5 days, the plated cells were treated with 6 μM CHIR99021 (CHIR, 034–23103, Wako) in RPMI-1640 medium (189–02025, Wako) containing 2% B27 supplement, minus insulin (RPMY/B27-insulin). Next day, the medium was changed without CHIR. Two days later, the medium was changed to that supplemented with 5 μM IWP-2 (030–24303, Wako). The culture medium was changed every other day with RPMI-1640 medium containing 2% B27 supplement (with insulin) until analysis.

QUANTIFICATION AND STATISTICAL ANALYSIS

Statistical analysis was performed using Student's *t* test, Dunnett's test or Fisher's exact test. p values <0.05 were considered statistically significant. *, ** or *** in the graphs indicate $p < 0.05$, $p < 0.01$ or $p < 0.001$, respectively. The specific test being used as well as the nature of the replicates are stated in the figure legends.

Cell Reports Methods, Volume *3*

Supplemental information

Efficient selection of knocked-in pluripotent stem cells using a dual cassette cellular elimination system

Koji Nakade, Satomi Tsukamoto, Kenichi Nakashima, Yuri An, Iori Sato, Jingyue Li, Yuzuno Shimoda, Yasuko Hemmi, Yoshihiro Miwa, and Yohei Hayashi

Figure S1

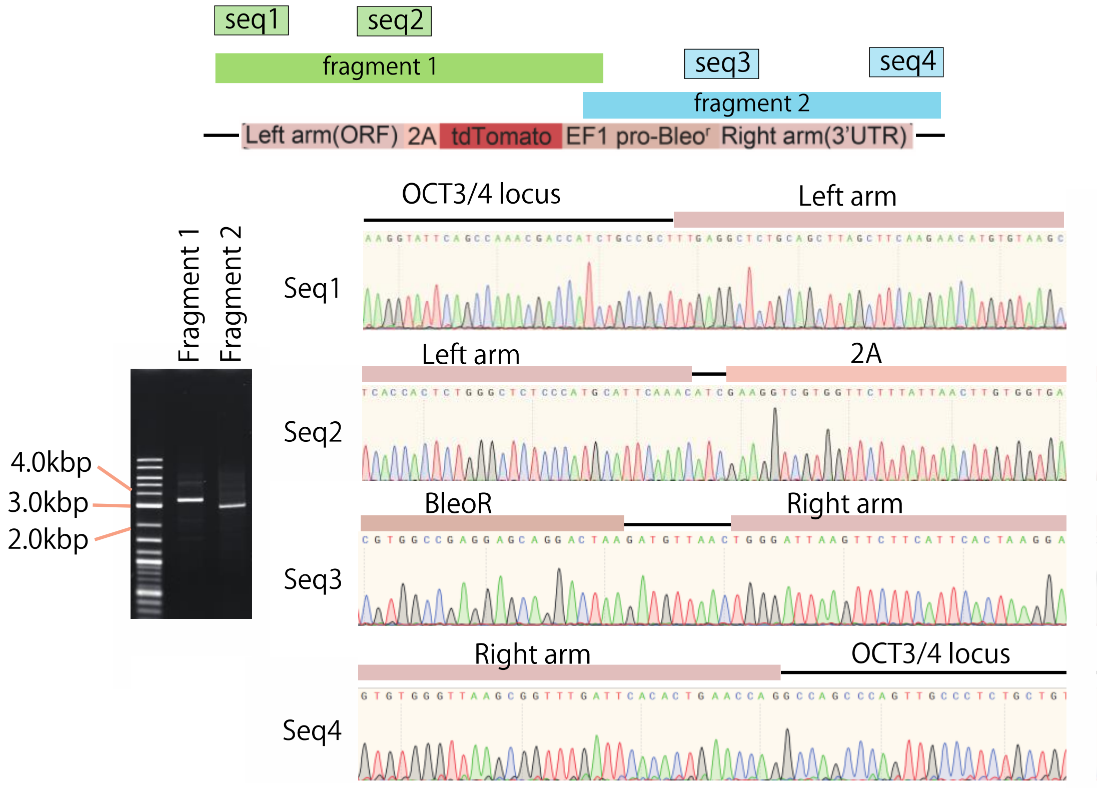

Figure S1. The representative sequencing schemes and results of *OCT4* genomic and knock-in alleles in a OCT3/4-TEZ(tk) clone, related to Figure 1.

Figure S2

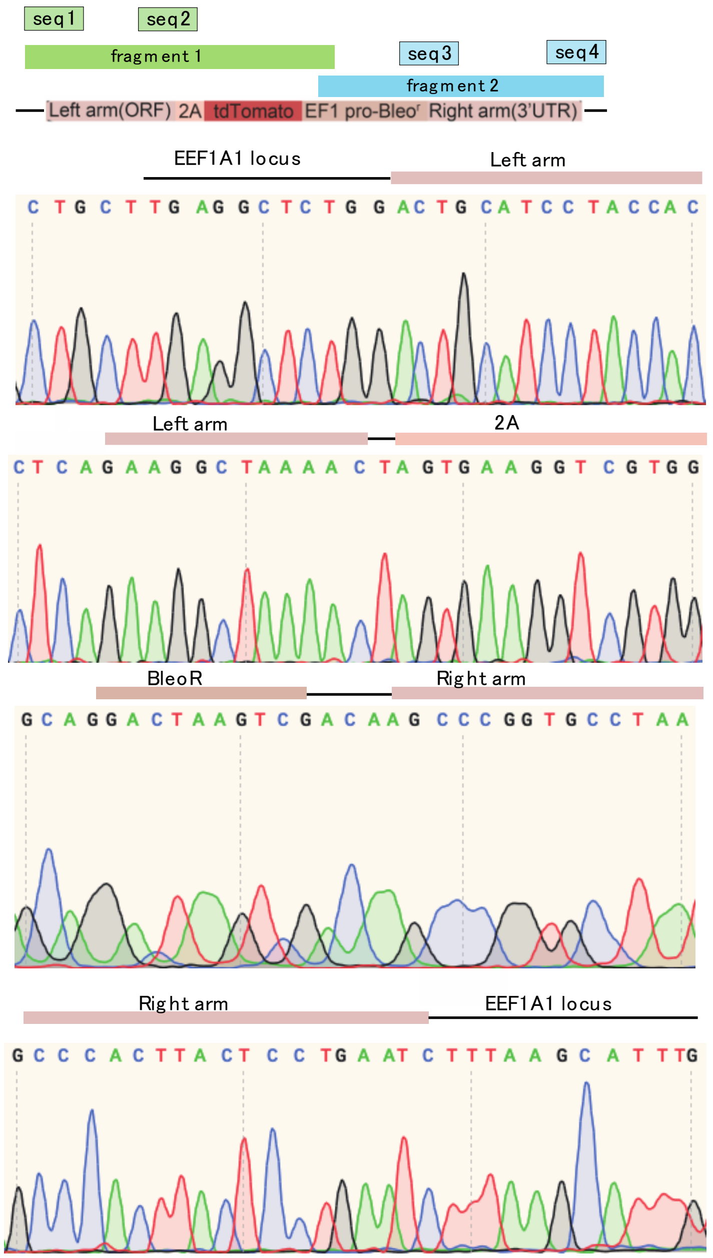

Figure S2. The representative sequencing schemes and results of *EEF1A1* genomic and knock-in alleles in a EEF1A1-TEZ knock-in clone, related to Figure 3.

Figure S3

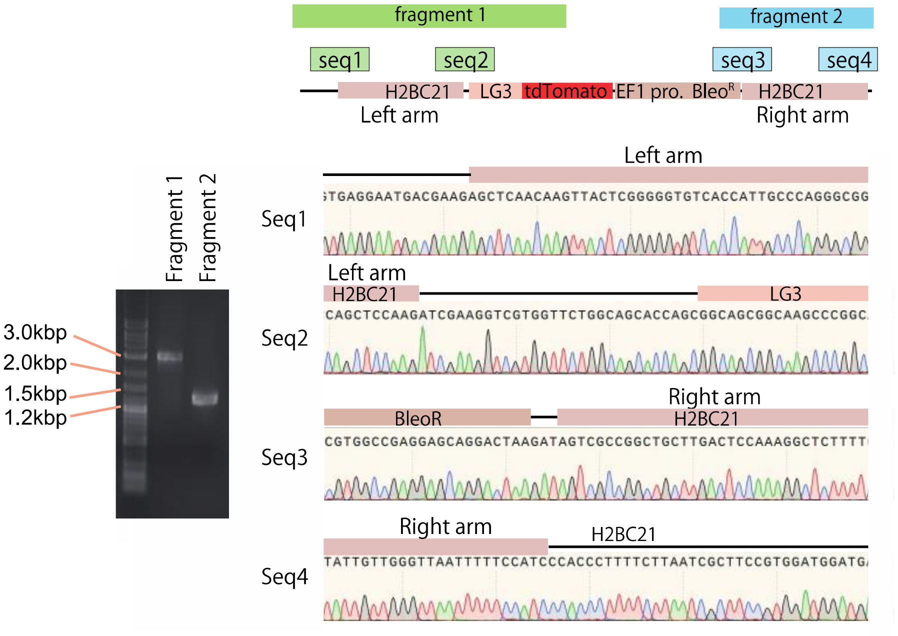

Figure S3. The representative sequencing schemes and results of *H2B* genomic and knock-in alleles in a H2B-TEZ knock-in clone, related to Figure 4.

Figure S4

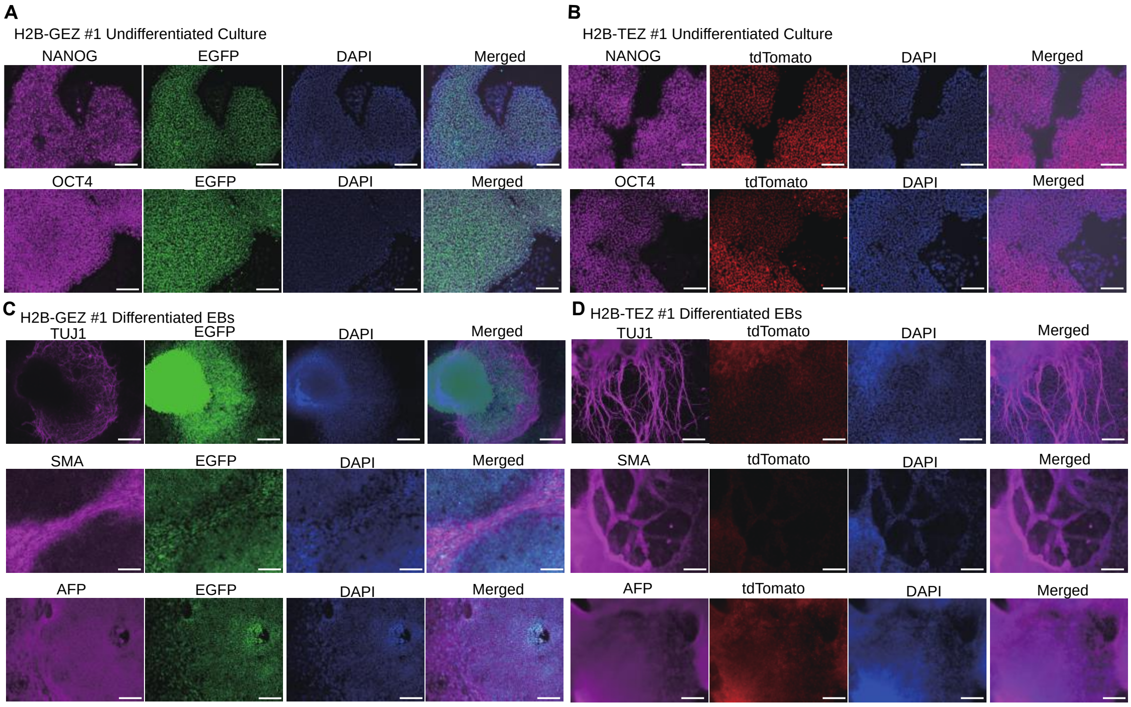

Figure S4. Self-renewal and pluripotency of H2B-GEZ #1 and H2B-TEZ #1, related to Figure 4. (A, B) Expression of self-renewal markers of hiPSCs, OCT3/4 and NANOG shown in purple. H2B-GEZ #1 and H2B-TEZ #1 were used. DAPI was used to stain nuclei (blue). EGFP or tdTomato were co-stained in green or red, respectively. Scale bars 100 µm. (C, D) Pluripotency in H2B-GEZ #1 and H2B-TEZ #1 was evaluated with EB formation assay. Immunocytochemistry of TUJ1 (ectoderm marker), alpha-SMA (mesoderm marker), and AFP (endoderm marker) in EB samples were shown in purple. DAPI was used to stain nuclei (blue). EGFP or tdTomato were co-stained in green or red, respectively. Scale bars, 100 µm.

Supplemental Table S1. The list of sequences of DNA primers or synthetic oligomers used in this study, related to STAR Methods.

Name	Sequence (5' -> 3')	Name	Sequence (5' -> 3')
gOCT3/4_F	CACCGAACTTAATCCCAAAAACCC	gOCT3/4_R	AAACGGGTTTTTGGGATTAAGTTC
gEEF1A1_F	CACCGAGAAACCTGSTGTGTTCCTT	gEEA1A1_R	AAACAAGGAACACACAGGTTTCTC
gISL1_F	CACCGATCATTGAATAGCATATAC	gISL1_R	AAACGTATATGCTATTCAATGATC
gH2B_F	CACCGACTCGAGCGAGCGAGCGCC	gH2B_R	AAACGGCGCTCGCTCGCTCGAGTC
gMYH7_F	caccTGTTACACAGGCTCCAGCAT	gMYH7_R	aaacATGCTGGAGCCTGTGTAACA
OCT3/4 LA-f	ATGGAAAAACGCCAGTTGAGGCTCTGCAGCTTA	OCT3/4 RA-r	AAAACGACGGCCAGTGCTGGTTCAGTGTGAATC
p15AoriF	TATAGTCCTGTCGGGTTTCG	OCT3/4 LA-r	AGAACCACGACCTTCGATGTTTGAATGCATGGGAGAG
OCT3/4 RAf	AAGCCCGGTGCCTAATCTAGATTCTAGGAATGGGGGACAGGG	p15AoriR	AACCCGACAGGACTATA
OCT3/4KI_Pr399	CAACTGCGTGCACTTCGTGG	OCT3/4KI_chkRA-r2	AGCAGAGGGCAACTGGGCTG
OCT3/4WT+KI_ RAf-HpaI	GTTAACTGGGATTAAGTTCTTCATTCACTA		
EEF1A1 LA-f	ATGGAAAAACGCCAGCCGTAAGGATGGCAATGCCAGT	EEF1A1 RA-r	GACGGCCAGTGAATTGGAGTAAGTGGGCTATTACACCTG
EEF1A1 RA-f	AAGCCCGGTGCCTAATCTAGATGTGTTCCTTTGGTCAACA	EEF1A1 LA-r	AGAACCACGACCTTCACTAGTTTTAGCCTTCTGAGCTTTC
2Af-1-16	GAAGGTCGTGGTTCT	Bleo-R inf-puro	TTAGGCACCGGGCTTGTCGACTTAGTCCTGCTCCTCG
H2B LA-f	ATGGAAAAACGCCAGCTCAACAAGTTACTCGGGGGTGTCA	H2B LA-r	TCCTCGGTACCAGAACCACGACCTTCGATCTTGGAGCTGGTGTACTTGGT
H2B RA-f	GTTCTGGTACCGAGGAGCAGGACTAAGATAGTCGCCGGCTGCTTGACTCCA	H2B RA-r	AAAACGACGGCCAGTGGATGGAAAAATTAACCCAACAATAT
M13RV	CAGGAAACAGCTATGAC	tdTom-r	CTCCTCGCCCTTGCTCACCAT
chk*ISL1* LA-f	TACCTGGAGGGCATTTGCTTT	*ISL1* LA-r	AGAACCACGACCTTCTGCCTCAATAGGACTGGCTAC
chk*ISL1* RA-r	GGCGCTTTGATTTCACATA	BleoR-f	GCACTTCGTGGCCGAGGAGCAGGAC
GFP-f	ATGGTGAGCAAGGGCGA	GFP-r	GGGTCGACCTTGTACAGCTCGTCCA
*H2BC21*_genotype_F	AGGGCGGGACTTGGCAGTTTAGAG	*H2BC21*_genotype_R	GTAAGTGCCTCGCAGTGACCTGGG
hsv-tk_genotype_F	TGGCTTCGTACCCCTGCCATCAAC	hsv-tk_genotype_R	AGCACCTGCCAGTAAGTCATCGGC
hsv-tk_F3	GCTTGCCAATACGGTGCGGT	hsv-tk_Pr949	CTTTTTATTGCCGTCATAGCG
ISL1 RA-f3	CCCTGTTGGAGAAAGTGGGA	*ISL1* RA-r3	CCACCTTCCTGAAAGGACTCT
MYH7 LA-f	tatagcatgaacagtGTCCAAGTTCCGCAAGGTGCAG	*MYH7 LA-r*	agaaccacgaccttcCTCCTCATTCAAGCCCTTTTGA
MYH7 RA-f	aagcccggtgcctaaCTGGAGCCTGTGTAACAGCT	*MYH7 RA-r*	aaaacgacggccagtACTCTTTCCTCCACCTCCTGGCC
chkMYH7 LA-r	CAGATCAAGATGTGGCAAAGCT	*chkMYH7 RA-r*	ACTCCAAAGGGGCTGAACTTG
ckOct3KI-LarmoutF	ctcaccctgggggttctatttggtg	*ckOct3KI-RarmoutR*	TAAGGGAAAACAGATCTATTGCCAT
EF1a-M_R	ACTTCCAACCCGAAGCTCG	*BGH_F2*	CTGTGCCTTCTAGTTGCCAG
tdTomato hindge-r	gttgttgtcctcggaggaggc	*ZeoR_F1*	ACGAGCTGTACGCCGAGTGG

Supplemental Table S2. The list of DNA plasmids used in this study, related to STAR Methods.

Plasmid Name	Reference number
pCRtk2x2-ISL1-TEZ	RDB18669, RIKEN BRC
pCRtk2x2NN	RDB18670, RIKEN BRC
pX330-gIsl1	RDB18671, RIKEN BRC
pUC-TEZ	RDB18672, RIKEN BRC
pUCtk2x2	RDB19569, RIKEN BRC
pX459-gEEF1A1	RDB19570, RIKEN BRC
pUCtk2x2-EEF1-TEZ	RDB19571, RIKEN BRC
pUCtk2x2-OCT3/4-TEZ	RDB19572, RIKEN BRC
pUC-2xLG3-GEZ	RDB19573, RIKEN BRC
pUC-2xLG3-TEZ	RDB19574, RIKEN BRC
pUCtk2x2-UAS	RDB19575, RIKEN BRC
pUC2x2Tk-H2B	RDB19576, RIKEN BRC
pUC2x2Tk-H2B-GEZ	RDB19577, RIKEN BRC
pUC2x2Tk-H2B-TEZ	RDB19578, RIKEN BRC
pUCtk2x2-MYH7-TEZ	RDB20120, RIKEN BRC
pX330-gH2B	RDB19579, RIKEN BRC
pX330-gOCT3/4	RDB19580, RIKEN BRC
pX330-gMYH7	RDB20121, RIKEN BRC
pUC-OCT3/4-TEZ	RDB19586, RIKEN BRC
pX330	#42230, Addgene
pX459	#62988, Addgene
pEGFP-N1	#6085-1, Clontech
